# UTP – Gated Signaling Pathways of 5-HT Release from BON Cells as a Model of Human Enterochromaffin Cells

**DOI:** 10.3389/fphar.2017.00429

**Published:** 2017-07-13

**Authors:** Andromeda Liñán-Rico, Fernando Ochoa-Cortes, Alix Zuleta-Alarcon, Mazin Alhaj, Esmerina Tili, Josh Enneking, Alan Harzman, Iveta Grants, Sergio Bergese, Fievos L. Christofi

**Affiliations:** ^1^Department of Anesthesiology, The Wexner Medical Center at The Ohio State University, Columbus OH, United States; ^2^Molecular Virology, Immunology and Medical Genetics, The Wexner Medical Center at The Ohio State University, Columbus OH, United States; ^3^Department of Surgery, The Wexner Medical Center at The Ohio State University, Columbus OH, United States

**Keywords:** EC cells, calcium, purinergic signaling, UTP, 5-HT, P2Y_4_, P2Y_6_

## Abstract

**Background:** Enterochromaffin cells (EC) synthesize and release 5-HT and ATP to trigger or modulate gut neural reflexes and transmit information about visceral/pain sensation. Alterations in 5-HT signaling mechanisms may contribute to the pathogenesis of IBD or IBS, but the pharmacologic or molecular mechanisms modulating Ca^2+^-dependent 5-HT release are not understood. Previous studies indicated that purinergic signaling via ATP and ADP is an important mechanism in modulation of 5-HT release. However, EC cells also respond to UTP and UDP suggesting uridine triphosphate receptor and signaling pathways are involved as well. We tested the hypothesis that UTP is a regulator of 5-HT release in human EC cells.

**Methods:** UTP signaling mechanisms were studied in BON cells, a human EC model, using Fluo-4/Ca^2+^imaging, patch-clamp, pharmacological analysis, immunohistochemistry, western blots and qPCR. 5-HT release was monitored in BON or EC isolated from human gut surgical specimens (hEC).

**Results:** UTP, UTPγS, UDP or ATP induced Ca^2+^oscillations in BON. UTP evoked a biphasic concentration-dependent Ca^2+^response. Cells responded in the order of UTP, ATP > UTPγS > UDP >> MRS2768, BzATP, α,β-MeATP > MRS2365, MRS2690, and NF546. Different proportions of cells activated by UTP and ATP also responded to UTPγS (P2Y_4_, 50% cells), UDP (P2Y_6_, 30%), UTPγS and UDP (14%) or MRS2768 (<3%). UTP Ca^2+^responses were blocked with inhibitors of PLC, IP3R, SERCA Ca^2+^pump, La^3+^sensitive Ca^2+^channels or chelation of intracellular free Ca^2+^ by BAPTA/AM. Inhibitors of L-type, TRPC, ryanodine-Ca^2+^pools, PI3-Kinase, PKC or SRC-Kinase had no effect. UTP stimulated voltage-sensitive Ca^2+^currents (I_Ca_), V_m_-depolarization and inhibited I_K_ (not I_A_) currents. An I_Kv_7.2/7.3 K^+^ channel blocker XE-991 mimicked UTP-induced V_m_-depolarization and blocked UTP-responses. XE-991 blocked I_K_ and UTP caused further reduction. La^3+^ or PLC inhibitors blocked UTP depolarization; PKC inhibitors, thapsigargin or zero Ca^2+^buffer did not. UTP stimulated 5-HT release in hEC expressing TPH1, 5-HT, P2Y_4_/P2Y_6_R. Zero-Ca^2+^buffer augmented Ca^2+^responses and 5-HT release.

**Conclusion:** UTP activates a predominant P2Y_4_R pathway to trigger Ca^2+^oscillations via internal Ca^2+^mobilization through a PLC/IP_3_/IP3R/SERCA Ca^2+^signaling pathway to stimulate 5-HT release; Ca^2+^influx is inhibitory. UTP-induced V_m_-depolarization depends on PLC signaling and an unidentified K channel (which appears independent of Ca^2+^oscillations or I_ca_/VOCC). UTP-gated signaling pathways triggered by activation of P2Y_4_R stimulate 5-HT release.

## Introduction

Enterochromaffin cells (EC) synthesize and release 5-HT, ATP and other mediators involved in gut neural reflexes and transmission of information about visceral/pain sensation (Kellum et al., 1999; Kim et al., 2001a; Raybould et al., 2004; Cooke and Christofi, 2006; Christofi, 2008). While EC cells release large quantities of endogenous 5-HT and exogenous 5-HT does stimulate gut motility, the mucosa and release of 5-HT from the mucosa are not required for *in vitro* peristalsis in the guinea-pig distal colon (Spencer et al., 2011) or *in vivo* intestinal transit of content (Yadav et al., 2010). However, abnormal regulation of 5-HT occurs in gastrointestinal disorders and inflammatory bowel diseases (IBD), where 5-HT signaling may represent a key mechanism in the pathogenesis of intestinal inflammation (Mawe and Hoffman, 2013; Liñán-Rico et al., 2016). Emerging evidence suggests that alterations in 5-HT release or handling mechanisms may contribute to IBD, Irritable Bowel Syndrome (IBS) and the diarrhea associated with bacterial toxin enterocolitis. Abnormal 5-HT signaling has also been implicated in diverticular disease, celiac disease, and colorectal cancer (Crowell, 2004; Galligan, 2004; Gershon, 2004; Kordasti et al., 2004; O’Hara et al., 2004; Manocha and Khan, 2012). Yet, the basic mechanisms regulating 5-HT release in human EC cells (hEC) are poorly understood. To understand the basis of these gastrointestinal disorders, it is necessary first to better understand how 5-HT release is regulated at cellular and molecular levels.

Enterochromaffin cells have chemo- and mechanosensitive elements that detect changes in force or contents of the intestinal lumen during peristalsis (Kim et al., 2001a; Christofi, 2008), the basic reflex underlying all motility patterns. The human BON cell line is a useful model to study chemosensation and mechanosensation, receptor regulation, post-receptor signaling pathways and physiological regulation of 5-HT release (Kim et al., 2001a,b, 2007; Cooke et al., 2003; Christofi et al., 2004a; Germano et al., 2009; Liñán-Rico et al., 2013).

Recent studies have employed freshly isolated hEC after acute isolation (Dammen et al., 2013) or in short term culture (Raghupathi et al., 2013) to study 5-HT release. However, the gold-standard for purinergic signaling studies remains the BON (EC) cell line since most of our knowledge of ATP (nucleotide) regulation of EC/5-HT signaling comes from these cells. A stable human cell line that is well characterized is appropriate for detailed mechanistic studies. Native hEC isolated from surgical specimens can be used to confirm key observations.

Purine receptors are broadly divided into nucleoside (P1, for adenosine) and nucleotide receptors (P2, for ATP, ADP, UTP and UDP). P2 is subdivided into P2X channel receptor (P2X_1-7_) and G-protein coupled receptor (P2Y_1,2,4,6,11-14_) families (Khakh et al., 2001; Kügelgen, 2006). Purinergic transmission occurs in the human enteric nervous system (Wunderlich et al., 2008; Liñán-Rico et al., 2015) and is known to act at all levels of gut secretory and motility reflexes (Burnstock, 2008; Christofi, 2008). Purinergic receptors are sensitive to mucosal inflammation and are emerging as potential novel therapeutic targets for GI diseases and disorders (Ochoa-Cortes et al., 2014).

Of particular interest is the role of purinergic signaling in EC cells. We could show that mechanical stimulation of the mucosa releases ATP that is required for triggering secretomotor reflexes (Christofi et al., 2004b; Cooke et al., 2004). Adenosine, a metabolite of ATP, is an important autoregulatory modulator of Ca^2+^-dependent 5-HT release (Christofi et al., 2004a). Our previous studies showed that purinergic signaling is an important mechanism in the modulation of 5-HT release. ATP is a critical determinant of mechanosensation and 5-HT release via autocrine activation of slow stimulatory P2Y_1_, inhibitory P2Y_12_ purinergic pathways, and fast ATP-gated P2X_3_-channels. Down-regulation of P2X_3_-channels (or alterations in A_2*B*_) is postulated to mediate abnormal 5-HT signaling (Liñán-Rico et al., 2013). The cognate ligands for these receptors are ATP and adenosine-5’-diphosphate (ADP). However, it is evident from our early observations that UTP and UDP, uridine nucleotides, can elicit Ca^2+^ oscillations in human BON (EC) cells. This suggests that a uridine nucleotide receptor is involved in Ca^2+^ oscillations leading to serotonin release in hEC. Candidate receptors are P2Y_2_, P2Y_4_ and P2Y_6_ receptors.

*We sought to test the novel hypothesis that UTP is a regulator of Ca^2+^ dependent 5-HT release in hEC by activating P2Y – uridine nucleotide receptors*. In light of the potential for targeting such receptors in GI diseases, we thought it would be important to identify the receptor(s), post-receptor, ionic and molecular signaling mechanisms linked to Ca^2+^ oscillations and 5-HT release. Collectively, our findings indicate that several uridine nucleotide receptors are linked to Ca^2+^oscillations, membrane depolarization, modulation of ionic currents and 5-HT release. Post-receptor signaling mechanisms include a GPCR/Gq/IP_3_-IP3R –Ca^2+^ signaling pathway, Ca^2+^ influx, SERCA, I_Kv 7.1,7.2/7.3_ and VOCC (I_ca_). Data support the hypothesis that UTP activates P2Y_4_ and P2Y_6_ receptors to modulate a GPCR/Gq/IP_3_-IP3R –SERCA Ca^2+^ signaling pathway leading to 5-HT release. Effect of UTP on membrane depolarization was not linked to a Ca^2+^ dependent 5-HT release.

## Materials and Methods

### IRB Approval

Studies conducted in hEC were carried out in accordance with the Declaration of Helsinki and approved by an Institutional Review Board (IRB) ethics committee at The Ohio State University. The protocol was # 2012H0231. Patient consent was obtained for each human subject and surgical tissue that is otherwise discarded by pathology was used to isolate hEC for studies on 5-HT release, or western blot analysis in human mucosa for P2Y receptors. Eight Roux-en-Y surgical specimens and 3 colonic specimens were collected for our studies. Colonic surgical specimens were obtained from patients undergoing a left colectomy for polyps/adenomas from the descending colon.

### Chemicals

Stock solutions of all drugs were prepared as per vendor indications, aliquoted and kept at -20°C (or -80°C) until used. ATP, UTP, UDP were from Sigma–Aldrich (St. Louis, MO, United States) and UTPγS, thapsigargin, U73122, U73343, GF109203X, α,β-MeATP, MRS compounds and XE-991 dihydrochloride were from TOCRIS Bioscience (Bristol, United Kingdom). Poly-D-lysine, Laminin, HBSS, DMEM: F12 medium were purchased from Life Science Technologies (Carlsbad, CA, United States). All other substances were purchased from Sigma–Aldrich (St. Louis, MO, United States).

### Cell Culture

#### Bon Cell Culture

BON cells were a gift from C.M. Townsend Jr (University of Texas, Galveston, TX, United States). Clone No. 7 was highly enriched with 5-HT. The cells were seeded on No. 0 cover slips (MatTek, Corp., Ashland, MA, United States) at a density of 5 × 10^4^ cells for calcium experiments and 5 × 10^5^ cells per well in 24-well culture plates (Corning-Costar Corp., Corning, NY, United States) for 5-HT release experiments. For patch clamp experiments cells were plated (6 × 10^3^) as droplets onto cover-slips pre-coated with Laminin/poly-D-Lysine (20 μg/ml each) and left to settle 2 h at 37°C. Cells were grown in Dulbecco-modified Eagle medium–nutrient mixture F-12 (1:1), supplemented with 10% fetal calf serum, 100 IU/mL penicillin, and 100 mg/mL streptomycin (Life Technologies, Grand Island, NY, United States). Cells were grown in a humidified atmosphere of 95% air and 5% CO_2_ at 37°C until reaching 70–90% confluence for calcium experiments (4–8 days after being seeded) and 48 h for 5-HT release. Passage numbers were from 29 to 66.

#### Human EC Culture

Human EC cells were isolated from mucosa of intestinal surgical specimens as described by Raghupathi et al. (2013). Mucosa was carefully scraped with bent forceps and collected in a 50 ml tube. The cells were washed with physiological buffer containing (in mM): 140 NaCl, 5 KCl, 2 CaCl_2_, 1 MgCl_2_, 10 HEPES, 5 D-glucose, pH 7.4. After 10 min centrifugation at 1000 rmp (4°C) the tissue was placed in digestion buffer containing 0.05% Trypsin-EDTA and 1 mg/ml collagenase A made in physiological buffer. Digestion was done at 37°C for 30 min with continuous agitation and triturating the cells every 10 min using a 5 ml pipette to pass the cells 20 times back and forth until no large pieces were visible. Enzymatic reaction was stopped by adding equal volume of DMEM with 10% FBS, 1% glutamine and 1% penicillin/streptomycin. Cell suspension was filtered through 100, 70 and 40 μm cell strainers into a 50 ml tube and centrifuged at 800 × *g* for 5 min. Pellet was re-suspended in 4 ml of growth medium and layer onto a Percoll density gradient prepared in NaCl. EC cells were harvested at a density of 1.07 g/L after centrifugation at 1,100 × *g* for 15 min. Cells were collected using a transfer pipette with a thin tip and rinsed with fresh media. Cells were re-suspended in 1 ml of complete DMEM + 10% FBS and 1.2 mM insulin, 68.7 nM transferrin, and 38.7 nM sodium selenite.

### Ca^2+^ Imaging

BON cells were loaded with 2 μM Fluo-4/AM (Molecular Probes, Eugene, OR, United States) in DMEM for 20 min in a 95% air and 5% CO_2_ incubator at 37°C. The Ca^2+^ response was monitored using a modified-Zeiss LSCM 410/REN laser scanning confocal imaging system. Cells were perfused at 6 ml/min with oxygenated Krebs solution (**Table 1**). A “solution inline heater” (Warner Instruments, Inc., Hamden, CT, United States) was used to maintain the perfusion temperature at 36.5 ± 0.5°C. Cells were imaged through a 40× oil immersion apofluor objective (numerical aperture 1.3, working distance = 170 μm). Ca^2+^ imaging was carried out using an Ar-Kr laser to excite the cells at 488 nm, and fluorescence emissions were passed through a FT510 dichroic mirror and collected through a photomultiplier tube equipped with a BP 505–550 filter, positioned in front of pinhole and light path. Time-series analysis of [Ca^2+^]_i_ was done at 1-s intervals.

**Table 1:** Standard solutions composition.

Reactive	Standard solutions (mM)					
	
	Krebs’	C-clamp Bath	C-clamp Electrode	ICa^2+^ Bath	ICa^2+^ Electrode	Potassium currents
NaHCO3	14.4					
NaH2PO4	1.35					
NaCl	120	130				
Glucose	12.7	5		5		1
KCl	6	5	30			5
CaCl_2_	2.5	2	2			1
MgCl_2_	1.35	1	1	2	4	1
HEPES		10	10	10	10	2
K-Gluconate			110			
NMDG				95		140
TEA-Cl				35		
BaCl_2_				10		
4-AP				1		
Cs-Gluconate					130	
EGTA					10	
ATP-Mg					4	
GTP-Na					0.3–0.5	
CdCl_2_						0.1
pH		7.3–7.4 NaOH	7.25 KOH	7.3–7.4 HCl	7.25 CsOH	7.3–7.4 HCl


Some experiments were performed using the Nikon FN1 Electrophysiology Microscope imaging system equipped with an Andor iXON Ultra camera (11 bit and 16 bit) for real-time calcium imaging; the camera is able to capture images at 55 frames/s. The system also includes the following components: A CFI Fluor 40× Water immersion objective lens (NA 0.8 WD 2 mm) was used with a long working distance achromatic condenser: NA 0.56 with adjustable diaphragm graduated in NA working distance 9.6 mm and a 6 place turret with fiber based light source (D-FI Universal EPI-Fluor Illuminator), Fluo-4 cube and NIS Elements Analysis 4.13 (Advanced calcium imaging analysis software).

Cells were perfused at 2 ml/min using a peristaltic pump, and drugs were applied by perfusion for 2 min (unless otherwise specified). The lag between drug application and response is due to time lag for drug to reach the desired concentration in the chamber at the perfusion rate and volume of the chamber (∼1.5 ml).

### 5-HT Release

BON cells were washed with a Ringer-like solution: 120 mM NaCl, 6 mM CsCl, 1 mM MgCl_2_, 10 mM glucose, and 10 mM HEPES-Na and 1.5 mM CaCl_2_ (pH 7.4) containing 0.1% BSA. For 0 Ca^2+^ Ringer-like buffer, extracellular calcium was omitted and 0.5 mM EGTA was added. After pre-incubation for 30 min at 37°C, the buffer was removed and replaced with 0.6 ml Ringer-like solution that contained 10^-5^ M of alaproclate (5-HT uptake inhibitor) and pargyline (monoamine oxidase inhibitor) and the drug of interest (ATP or UTP). Cells were incubated for 30 min at 37°C and after that, 0.5 ml of the assay buffer was collected in a 1.5 ml tube containing 1% stabilizer (LDN, Nordhorn, Germany) and frozen at -80°C until the 5-HT assay was performed.

For 5-HT release in human EC cells, 1 × 10^5^ cells were centrifuged at 8 × g for 10 min/4°C, medium was removed and replaced with 0.2 ml EBSS buffer containing 1.8 mM CaCl_2_, 0.8 mM MgSO_4_-7H_2_O, 5.3 mM KCL, 26 mM NaHCO_3_, 117 mM NaCl, 1 mM NaH_2_PO_4_-H_2_O and 5.5 mM D-Glucose with 0.1% BSA, 10^-5^ M of alaproclate and pargyline. Cells were allowed to equilibrate for 2 h at 37°C. After incubation, the drugs were added (final volume of 204 μl) and cells were incubated for 30 min at 37°C. Cells were centrifuged 8 × *g* for 5 min/4°C and the supernatant was collected in a 1.5 ml tube containing 1% stabilizer (LDN, Nordhorn, Germany) and saved at -80°C. 5-HT release was measured by enzyme immunoassay using an ELISA kit (LDN, Nordhorn, Germany) according to the manufacturer’s instructions. The absorbance was measured at 450 nm and 5-HT concentration was determined from a standard curve.

### Western Blot

Cells were washed with cold PBS and lysed in lysis buffer containing: 50mM Tris–HCl, pH 7.4, 150 mM NaCl, 1 mM Na_3_VO_4_, 1 mM NaF, 0.25% sodium deoxycholate, 1% NP40, 1 mM EGTA, 1 mM PMSF and protein inhibitor cocktail from Sigma–Aldrich (P8340). Protein concentration was calculated with Bradford assay (Bio-Rad, Hercules, CA, United States). Eighty microgram of cell lysate (or 20 μg for human mucosa) was denatured at 90°C for 10 min in 1:1 volume of 2X Laemmli sample buffer (Bio-Rad, United States) and 8% 2-mercaptoethanol. Protein samples were separated on a 10% SDS–PAGE gel and transferred to a PVDF membrane (Bio-Rad, Hercules, CA, United States). After blocking in 5% non-fat dry milk buffer, the membranes were incubated overnight at 4°C with rabbit anti-P2Y_2_ (1:200; Alomone, Jerusalem, Israel, APR-010), anti-P2Y_4_ (1:800; Alomone, APR-006), anti-P2Y_6_ (1:600; Alomone, APR-011), TPH1 (1:1000; Novus Biologicals, Littleton, CO, United States, NB110-57629) or anti-mouse β-actin (1:1000; Santa Cruz Biotech, Dallas, TX, United States, sc-47778) in blocking solution. Membranes were washed and incubated with the HPR-conjugated secondary anti-rabbit or anti-mouse antibody (1:1000; Cell Signaling Technology, Danvers, MA, United States).

### Immunochemical Labeling

Cells were fixed in 4% PFA for 20 min and washed with PBS. Cells were blocked for 30 min with 10% normal donkey serum, 0.5% Triton X-100 in PBS. Primary antibody incubation was done at 1:50 dilution for rabbit anti-P2Y_4_ (Alomone, Jerusalem, Israel) and 1:20 dilution for mouse anti-5-HT (Dako, Carpinteria, CA, United States, M0758). After washing, incubation with Alexa-fluor 488 or 568 secondary antibody at 1:400 dilution (Life Tech, Cedarhurst, NY, United States) was done for 30 min at room temperature, protected from light.

### RNA Isolation and Quantitative Real-Time PCR (qRT-PCR)

RNAs were extracted using TRIzol (Invitrogen, Buffalo, NY, United States). They were subsequently subjected to DNase digestion (Turbo-DNase Ambion – Life Technologies, Grand Island, NY, United States). Two micrograms per sample RNA was used to prepare the cDNA utilizing High-Capacity cDNA Reverse Transcription Kit (Random priming) with RNase Inhibitor (Life Technologies, Grand Island, NY, United States). Quantitative RT-PCRs were performed using the TaqMan Gene Expression Assays from Applied Biosystems – Life Technologies (Grand Island, NY, United States): Hs00267404_s1 for P2Y_4_ and Hs00366312_m1 for P2Y_6_. Quantitative RT-PCRs (100 ng of cDNA per 20-μL amplification reaction) were performed in triplicates. Gene expression levels were quantified using the ABI Prism 7900HT Sequence detection system (Applied Biosystems). Relative expression was calculated using the comparative *C_t_* method. Values were normalized using *OAZ1* Gene Expression assay (TaqMan assay Hs00427923_m1).

### Patch-Clamp Experiments

Recordings were made from BON cells after 4 days of culture on a SliceScope Pro upright microscope (Scientifica-Olympus, Uckfield, United Kingdom). Membrane potential was assessed using perforated patch with amphotericin B, 300 μg/mL. Thin walled glass-capillaries (Warner Instruments) were used in electrode fabrication with a horizontal micropipette puller P-97 (Sutter Instrument Co., Novato, CA, United States). Pipette resistance was 2–5 MΩ when immersed in bath solution. Cell signals were amplified and digitized by using Multiclamp 700B amplifier and Digidata 1440A converter, and stored using pClamp 10.3 software (Molecular Devices, Sunnyvale, CA, United States).

Experiments were performed at 34 ± 0.5°C and temperature was maintained using a single channel TC-324B temperature controller (Harvard Apparatus, Holliston, MA, United States). During experiments, cells were continuously perfused with bath external solution to an approximate rate of 2 mL/min with and without required testing compounds; UTP was applied for 2 to 3 min while changes in resting membrane potential were recorded. Other treatments or inhibitors were pre-incubated as indicated in specific experiments. Changes in Ri were assessed by applying hyperpolarizing current pulses of -10 mV at a frequency of 6 Hz. When necessary, cells were previously incubated with antagonists as required. Standard solutions are described in **Table 1**.

In current clamp experiments a liquid junction potential was calculated to be -10.8 mV, and was corrected for analyzing the data. I_Ca^2+^_ were recorded using whole cell patch-clamp and series resistance was compensated in every cell at least 70%; cells with a holding leak-current larger than -50 pA were rejected and no further leak correction was made. Once the Giga seal was formed and membrane ruptured in normal bath solution, cells were bathed 10 min with Cs^+^/Ba^2+^ solution to allow a complete exchange of solutions and internal medium dialysis. Ba^2+^ current was then assessed through voltage-operated Ca^2+^ channels (VOCC). Currents were stimulated giving 100 ms voltage steps from -70 to 40 mV with increments of 10 mV, every 5 s, from a holding potential (V_h_) of -80 mV and recorded at a sampling rate of 10 kHz and low pass filtered to 1 kHz. All currents were expressed as current density (pA/pF) and normalized to cell capacitance. Currents were changed to conductance to generate activation curves, plotted as *G*/*G*_max_ and fitted with a Boltzmann equation.

For voltage-dependent potassium currents (K_v_), normal pipette solution was used. Type A (I_A_) and type K (I_K_) voltage dependent potassium currents were separated by their inactivation properties. Total potassium currents were evoked with 500 ms voltage pulses ranging from -90 to +50 mV from a V_h_ of -100 mV (I_Tot_) and -40 mV (I_K_). I_K_ peak amplitude was measured at the end of the traces obtained in each voltage step and I_A_ was at the maximum peak (start of pulse) produced after withdrawing I_K_ from I_Tot_. Currents were normalized to cell capacitance and are shown as current density (pA/pF) after being fitted with a Boltzmann equation.

### Experimental Protocols

(1) We tested the effect of UTP, ATP and agonists with selectivity for P2Y_1_ (MRS2365), P2Y_2_ (MRS2768), P2Y_2/4_ (UTPγS), P2Y_6_ (UDP), P2Y_11_ (NF546), P2Y_14_ (MRS2690), P2X_7_ (BzATP) and P2X_1,2/3,3_ (α,β-MeATP) on Ca^2+^ responses. Drug concentrations were selected according to reported selectivity and EC_50_s in human cells (Abbracchio et al., 2006; Brunschweiger and Müller, 2006; Kügelgen and Bonn, 2008).
(2) A concentration-dependent Ca^2+^ response to UTP was determined in the range of 1 nM to 5 mM. Different concentrations of UTP were applied at 10 min intervals to allow sufficient time to washout and fully recover from the drug (as determined in preliminary experiments) (Brinson and Harden, 2001).
(3) Using this protocol, 3–4 different concentrations of drug could be applied to each cell to construct a full dose-response curve. It is possible to demonstrate a concentration-dependent effect in the same cell. However, it is not possible to determine the entire concentration response curve in each cell with reproducibility (i.e., possibly due to cell fatigue, desensitization, prolonged exposure to light to monitor Ca^2+^ responses, etc.). Therefore, analysis allowed determination of apparent EC_50_s, since a full concentration response curve could not be obtained in the same cell.
(4) We determined the profile of Ca^2+^ activity in response to consecutive applications of selective uridine nucleotide agonists (i.e., MRS2768, UTPγS, UDP) to identify the proportions of UTP-responsive cells with functional P2Y_2_R, P2Y_4_R and P2Y_6_R receptors.
(5) Determined the expression of uridine phosphate receptors by western blot, immunofluorescent labeling and/or qPCR analysis for selected receptors in BON, isolated human EC cells from intestinal surgical specimens, human mucosa and HT-29 cells.
(6) UTP effects of 5-HT release were analyzed in BON and human EC (hEC) cells.
(7) Ca^2+^ signaling pathways linked to UTP were determined using pharmacological inhibitors, receptor or channel blockers. Drugs were pre-incubated for 10–30 min depending on the drug. Paired data was obtained for all treatments (unless otherwise noted).
(8) Determined the effects of UTP on V_m_ depolarization and voltage-operated Ca^2+^channels (VOCCs) and voltage-activated potassium channels (K_v_ channels) by evaluating (i) The effect of UTP induced rise in [Ca^2+^]_i_ on V_m_ Depolarization of the EC cells. (ii) Determined if UTP activates voltage-operated Ca^2+^ channels (VOCCs) and induces I_ca_ currents in EC cells. (iii) Determined if UTP modulation of voltage-activated potassium currents (K_v_ currents: I_K_ and I_A_ type currents) are involved in the depolarization of EC cells.

### Statistical Analysis

Statistical analysis of data was performed in Graphpad Prism 6 (La Jolla, CA, United States). Paired, unpaired *t*-test, Fisher exact test and two ways ANOVA with Bonferroni post-tests were used when appropriate. All data are expressed as mean ± SEM. A *p* < 0.05 is considered significant.

## Results

### Pharmacology of Uridine Nucleotides in BON

**FIGURE 1:**
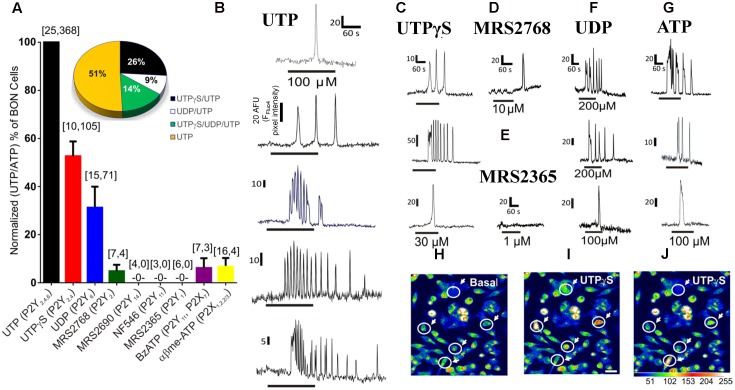
Pharmacology of purinergic receptors linked to Ca^2+^responses in BON cells. **(A)** Percentage of UTP (or ATP) responsive cells displaying Ca^2+^ responses to selective purinergic agonists. Ca^2+^ responses were normalized to % of UTP or ATP responsive cells, since same subset of cells were always responsive to both agonists; the first number inside the brackets represents the number of cultures and the second is the number of responsive cells. The Ca^2+^ response to agonists follows the order of UTP/ATP > UTPγS > UDP >> MRS2768 = BzATP = α,β-MeATP > MRS2365, MRS2690, NF546. Pie graph inset shows the response patterns to agonists obtained from consecutive applications of UTPγS, UDP and UTP in the same cells of a single experiment, *n* = 69 cells; repeated in *N* = 5 cultures. Representative Ca^2+^ transients are shown for **(B)** UTP, **(C)** UTPγS, **(D)** MRS2768, **(E)** MRS2365, **(F)** UDP and **(G)** ATP. Line below the transient indicates duration of drug application. Each of the drugs was perfused at the concentrations indicated in Supplementary Table 1 for 1 min followed by ATP (or UTP) for UTPγS, UDP and MRS2768 or MRS2365) perfusion with a 10–15 min washout between each drug application. **(H–J)** Representative pseudocolor images of Ca^2+^ responses to 30 μM UTPγS, plotted on a pixel intensity scale from 0 to 255. Responsive cells are circled with an arrow. Scale bar = 30 μm.

In the first series of experiments, we characterized the functional uridine nucleotide receptor(s) activated by the cognate ligand UTP in BON cells. The pharmacological effects of various purinergic agonists were evaluated in inducing Ca^2+^ responses in BON. With the exception of UTP, a suitable concentration of each selective agonist was used to evaluate activity at different receptors (Wihlborg et al., 2003; Jacobson et al., 2009; Jacobson and Linden, 2011). Data for agonists were normalized as a % of UTP (or ATP) responsive cells (**Figure 1A**). The same cells responded to UTP and ATP, and these agonists caused similar responses (Supplementary Figure 1). A subset of UTP/ATP responsive cells displayed UTPγS responses and fewer cells responded to UDP. MRS2768 was virtually ineffective and it only rarely gave a response (**Figure 1A**).

UTP (P2Y_2,4,6_), UTPγS (P2Y_2,4_), UDP (P2Y_6_) and ATP could evoke a single monophasic Ca^2+^ transient, or more often caused Ca^2+^ oscillations (**Figures 1B,C,F,G**). The P2Y_2_ agonist MRS2768 could only evoke a monophasic Ca^2+^ transient in a tiny subset of cells (**Figure 1D**). Agonists that did not evoke a Ca^2+^ response include a P2Y_14_ agonist MRS2690, NF546 (P2Y_11_) and MRS2365 (P2Y_1_, **Figure 1E**). BzATP and α,β-MeATP (P2X agonists) caused Ca^2+^ responses in less than 10% of UTP responsive cells (not shown). The percentage of the cell population with a Ca^2+^ response to agonists followed the order of those responding to UTP (and ATP) alone (∼50% cells) > UTPγS (and UTP, 26%; P2Y_4_) > UTPγS/UDP (and UTP, 14%; P2Y_4_, P2Y_6_) > UDP (and UTP, 9%; P2Y_6_) > MRS2768 (and UTP, ∼3%; P2Y_2_) = BzATP (P2X_7_) = α,β-MeATP (P2X_1,2/3,3_) > MRS2365 (P2Y_1_), MRS2690 (P2Y_14_), NF546 (P2Y_11_, < 1% each). Examples of pseudocolor images of Ca^2+^responses to UTPγS are shown in **Figures 1I,J** compared to Basal (control) in **Figure 1H**.

**FIGURE 2:**
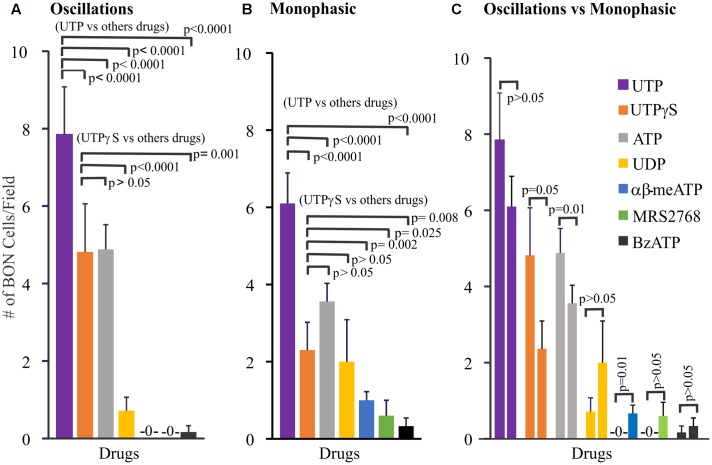
Profile of different Ca^2+^-responses evoked by agonists for purinergic receptors in human BON. Agonists elicit monophasic Ca^2+^ transients or Ca^2+^ oscillations. **(A)** Profile of agonist responses for Ca^2+^ oscillations. **(B)** Profile of agonist responses for monophasic Ca^2+^ transients. **(C)** Comparison of the number of cells displaying oscillations (first column) versus monophasic (second column) Ca^2+^ transients in response to each purinergic agonist. Poisson regression was used for statistical comparisons in **(A,B)**, and *t*-test in **(C)**. For this analysis only one drug at a time was tested in each cell culture. Concentrations of each drug are listed in Supplementary Table 1 (see results for more details). The average number of cells/field was 46 ± 8. The proportion of cells responding to UTP is ∼20%; for UTPγS, it was 11%.

Further pharmacological analysis tested whether monophasic and oscillatory responses are mediated by different receptors. In these experiments, only one drug at a time was tested in each cell culture. To do this we analyzed the pharmacological profile of selective agonists for oscillations (**Figure 2A**), monophasic responses (**Figure 2B**) and made comparisons between the profile of agonists between oscillations and monophasic responses (**Figure 2C**). Using Poisson regression analysis for numbers of cells/field responding to each agonist, the response profile for oscillations is UTP > UTPγS = ATP >> UDP > BzATP > α,β-MeATP, MRS2768, NF546. Supplementary Table 1 summarizes information on selectivity of each agonist, drug concentrations used, and responsive cells to each agonist [ATP, 100 μM; α,β-MeATP, 10 μM/30 μM; BzATP, μM/300 μM; UTPγS, 30 μM; UDP, 100 μM; MRS2768, 10 μM; NF546, 30 μM; MRS2365, 1 μM/5 μM; MRS2690, 0.5 μM]. For monophasic responses, the agonist response profile is UTP >> UTPγS = ATP = UDP > α,β-MeATP > BzATP = MRS2768. Direct comparisons between oscillations and monophasic responses for each agonist indicates that significant differences exist for UTPγS, ATP (more cells with oscillations) and α,β-MeATP (more cells with monophasic responses).

**FIGURE 3:**
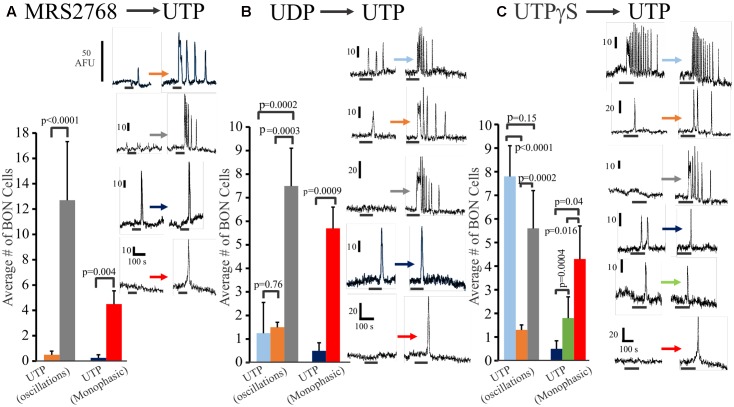
Oscillations or monophasic Ca^2+^ transients involve P2Y_4_ and P2Y_6_ receptors in human BON. Ca^2+^ responses to consecutive applications of **(A)** a P2Y_2_ agonist MRS2768, **(B)** a P2Y_6_ agonist UDP or **(C)** a P2Y_2,4_ agonist UTPγS followed by UTP (washout of 10 min, drugs tested in 4–5 separate cultures). The color of each column represents a particular type of response that is illustrated in representative Ca^2+^ transients at the right of each histogram. For example, in **(A)**, the gray bar represents cells with no response to MRS2768 and Ca^2+^ oscillations to UTP. The red bar represents cells with no response to MRS2768 and a monophasic Ca^2+^ transient to UTP. UTPγS in contrast to UDP more closely mimics responses to UTP in a significant proportion of cells.

In a separate set of experiments, we further explored the relative distribution of functional P2Y_2_, P2Y_4_ and P2Y_6_ receptors on BON cells in experiments where we sequentially tested the effects of (1) MRS2768 (P2Y_2_) followed by UTP (MRS2768 → UTP), (2) UDP → UTP, and (3) UTPγS → UTP. Data is summarized in **Figure 3**. Very few (<10%) UTP responsive cells had MRS2768 responses, and responses were monophasic only (**Figure 3A**). UTP causes both oscillations and monophasic responses. A small subset of UTP-responsive cells had UDP responses (both types), but most were monophasic responses, suggesting most oscillations were in response to P2Y_4_ receptor activation (**Figure 3B**). However, different subsets of cells responded to UTPγS/UTP with oscillations, UTP alone with oscillations, UTP alone with monophasic responses, UTPγS with monophasic/UTP with oscillations, UTPγS/UTP with monophasic responses (**Figure 3C**).

**FIGURE 4:**
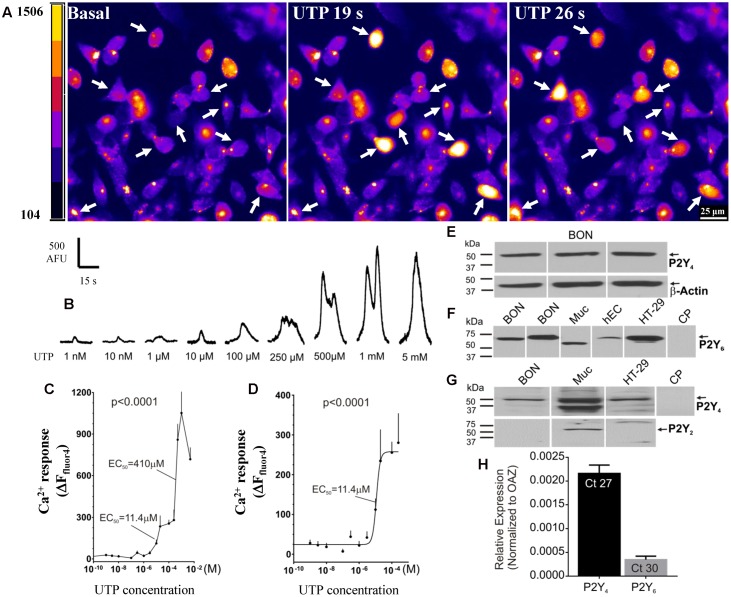
Function and molecular characteristics of uridine nucleotide receptors activated by UTP. **(A,B)** UTP elicits a concentration dependent Ca^2+^ response in BON. **(A)** Representative images of BON cells in response to 100 μM UTP perfusion. Cells responding to UTP with a Ca^2+^response are marked with an arrow; pseudocolor images based on pixel intensity (0–1500 arbitrary units). **(B)** Representative Ca^2+^ transients to separate applications of UTP in the concentration range of 1 nM to 5 mM. **(C)** Biphasic concentration-response curve to UTP for peak Ca^2+^ responses. ANOVA, *p* < 0.0001 (310 cells were analyzed). The apparent EC_50_ of the first phase of the response is 11.4 μM; the EC_50_ of the second phase is 410μM. **(D)** The first phase of the UTP concentration – response curve (0.1 nM–0.2 mM) is fitted with a logistic equation; symbols represent the net increase in Ca^2+^ response at each time point and the error bars are SEM (*n* = 15 or more cells at each concentration). **(E)** Representative western blots (WB) showing immunogenic bands for P2Y_4_R in BON cells. **(F)** Representative immunogenic bands for P2Y_6_R in BON, human mucosa (Muc) and isolated human EC cells from surgical specimens (hEC), and human HT-29 cells. Pre-absorption of the anti-P2Y_6_R antibody with its immunogenic peptide (CP) blocked the immunogenic bands. **(G)** P2Y_4_ is detected in BON, Muc and HT-29 cells. P2Y_2_R is only detected in the human mucosa and not BON or HT-29. **(H)** TaqMan PCR analysis showing the relative expression of P2Y_4_R and P2Y_6_R normalized to OAZ as 2ˆ(–ΔCt). The *C_t_* values are indicated inside each column, *N* = 6 cultures. BON cells for qPCR were tested at passage 44, and P2Y_4_ expression was >P2Y_6_ expression (see Results and Supplementary Figure 2).

UTP elicits a concentration-dependent Ca^2+^ response in BON cells in the range of 1 nM–5mM (**Figures 4A–D**). In this concentration range, a biphasic effect occurs with apparent EC_50_ values of 11.4 and 410 μM UTP (**Figure 4C**). The best-fit curve for the first phase of the response is plotted with a logistic equation in **Figure 4D**.

In summary, UTP activates multiple receptors in BON to evoke a Ca^2+^ response, but the predominant uridine nucleotide receptor operating in BON is the P2Y_4_ receptor. Subpopulations of responses to different agonists suggest that UTP, ATP and UDP can activate different combinations of purinergic receptors in different cells, and therefore, subtypes of EC cells can be distinguished by their functional expression of purinergic receptors.

### Expression of P2Y Receptors

Western blot analysis for P2Y_4_ receptors identified a protein with a molecular weight of ∼50 kDa in BON cells, EC cells, human mucosa, as well as human adenocarcinoid HT-29 epithelial cells, whereas immunogenic bands for P2Y_2_ were identified only in mucosa, and were absent from BON cells (**Figures 4E,G**). P2Y_6_ immunogenic bands were identified in BON cells, mucosa, HT-29 and human enterochromaffin cells (hEC) isolated from surgical specimens (**Figure 4F**). Immunogenic bands were eliminated by pre-absorption of antisera with CP (**Figures 4F,G**).

TaqMan PCR analysis for P2Y_4_ and P2Y_6_ (the two main P2Y receptors identified in pharmacological studies) indicated that mRNA expression for P2Y_4_ was much higher than P2Y_6_ in BON cells (**Figure 4H**). As shown in Supplementary Figure 2, mRNA expression level of P2Y_6_ is passage dependent in BON cells. Therefore, in passages <36, 64% of UTP responsive cells respond to UDP (P2Y_6_) whereas in passages >43 only 9% of UTP responsive cells, respond to UDP. Regardless of passage, ∼50% of UTP responsive cells respond to UTPγS (P2Y_4_). Our studies were conducted in cells with both functional receptors present.

**FIGURE 5:**
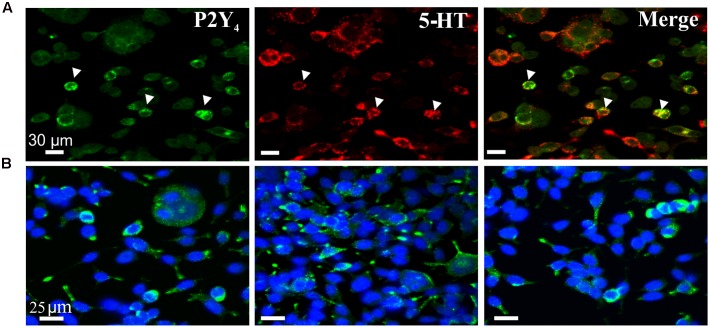
P2Y_4_R immunoreactivity in BON cells. **(A)** Co-labeling of P2Y_4_ receptors (green) with 5-HT (red) in BON cells. P2Y_4_ is co-localized in a subset of BON cells expressing 5-HT immunoreactivity; arrowheads, co-localization of P2Y_4_ and 5HT immunoreactivities. **(B)** BON cells labeled for P2Y_4_ immunoreactivity (green) are counter-stained with DAPI (blue). P2Y_4_ immunoreactivity is localized around the cell nucleus.

**FIGURE 6:**
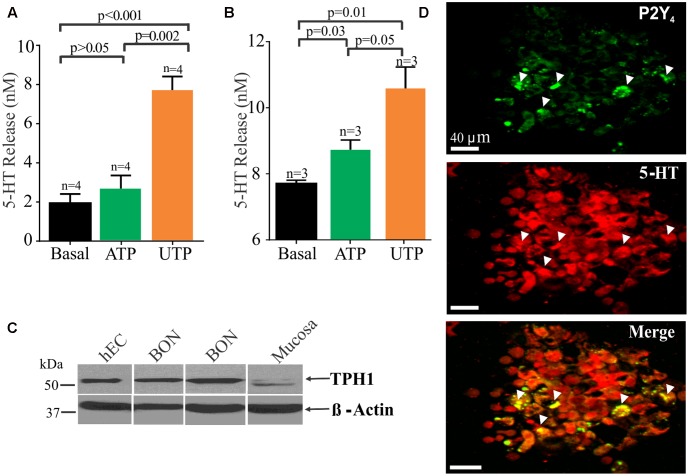
UTP stimulates 5-HT release in BON cells and human EC cells isolated from surgical specimens. **(A)** UTP stimulates 5-HT release in BON. **(B)** UTP stimulates 5-HT release in human EC cells; UTP and ATP are applied at 100 μM each. **(C)** Western blot showing TPH1 expression in human EC cells, BON cells and human mucosa; β-actin is used as a housekeeping gene. Samples were run in the same gel. **(D)** Co-labeling for P2Y_4_ and 5-HT immunoreactivity in human EC cells isolated from human surgical specimens.

The western blot expression of the P2Y_4_ receptor was confirmed by immunofluorescent labeling to show expression of P2Y_4_ immunoreactivity in BON cells co-expressing 5-HT immunoreactivity (**Figure 5**) and EC cells (**Figure 6**). Abundant P2Y_4_ immunoreactivity is expressed in the cells. Our study was not designed to detect whether the P2Y_4_ receptors were localized to the surface of the cells. This could be done by radioimmunoassay (Brinson and Harden, 2001) and confirmed by detailed z-stack confocal imaging experiments to determine whether P2Y_4_ is localized in the membrane or is also in the cytosol. P2Y_4_ receptors can undergo internalization upon activation, and receptors are expected to distribute in both membranes and inside the cell. Our pharmacological data support a functional role for the P2Y_4_ receptor.

### UTP Modulation of 5-HT Release

It is well known that EC cells are the main source of 5-HT of the entire body and as anticipated, UTP and ATP stimulated 5-HT release from BON cells (**Figure 6A**) or hEC (**Figure 6B**) isolated from surgical specimens (**Figures 6A,B**). However, UTP was more effective in stimulating 5-HT release than ATP at equimolar concentrations (100 μM each). Furthermore, immunoreactivity for P2Y_4_ receptors was also confirmed in hEC cells (**Figure 6D**) as shown to occur in BON cells (**Figure 5**). Western blot analysis identified the 5-HT synthesizing enzyme (TPH1) in both hEC and BON cells (**Figure 6C**).

In hEC cells identified by their 5-HT immunoreactivity and DAPI counter stain, 28.82 ± 12.43% of 694 cells express P2Y_4_ receptor immunoreactivity. Cells were counted in 20 random fields from cells isolated from jejunum surgical specimens in two patients. A caveat here is that this is a pilot analysis, and it may not represent the population response in human jejunum EC cells. It is important to point out that that P2Y_4_ receptors can undergo internalization after activation by a high enough concentration of UTP (Brinson and Harden, 2001), and the EC cell isolation procedure used is sufficient to mechanically activate the cells to release UTP (or other purines) and cause receptor internalization to varying degrees in the cells. Cells can take many hours to recover (i.e., up to 12 h). Apparently, there are both punctate and other staining distributions in different EC cells (probably representing localization at both cell surface and inside the cells). EC cells are sensitive to mechanical stimulation, and even changing the medium on the cells after plating can cause release of UTP (Lazarowski et al., 1997; Lazarowski and Harden, 1999) that can activate receptors.

### Ca^2+^ Signaling Pathways Activated by UTP

5-HT release is a Ca^2+^ dependent mechanism, and therefore, we sought to determine the post-receptor signaling pathways linked to UTP – Ca^2+^ responses. Comprehensive analysis was done with pharmacological inhibitors, receptor or channel blockers, and data is summarized in **Tables 2**, **3**. As well, given the complexity of the pathway, we decided to present the results for Ca^2+^ imaging and patch-clamp separately.

**Table 2:** Post-receptor signaling pathways linked to UTP-Ca^2+^ responses.

Drug (+UTP)	[μM]	Effect on UTP Ca^2+^ oscillations	Mechanism	*n*	*p*-value
0 Ca^2+^ EGTA	500	Augmented the average response	Ca^2+^ chelator	45	0.038^b^
BAPTA-AM	50	Blocked in 100% of the cells	Ca^2+^ chelator	23	<0.001^a^
U73122 vs. U743343	10	Blocked in 69% of the cells	PLC inhibitor	16	0.001^a^
		Average inhibition of 73%		23	0.0001^a^
La^3+^	100	Blocked in 100% of the cells	Ca^2+^ channel blocker	57	<0.0001^a^
2APB	100	Blocked in 100% of the cells	IP3r antagonist, inhibits SOC release	27	<0.0001^a^
Thapsigargin	1	Blocked in 100% of the cells	Ca^2+^ pump SERCA inhibitor	16	0.001^a^
RuRed	10	No effect	Ryanodine receptor blocker	12	NS^b^
Ly29400	10	No effect	PI3K inhibitor	28	NS^b^
GF109203X	1, 3	No effect	PKC inhibitor	10, 32	NS^b^
MRS1845	20	No effect	Inhibits store-operated Ca^2+^ entry	33	NS^b^
PP2	10	No effect	SRC kinase blocker	20	NS^b^
SKF96365	50	No effect	TRPC channel blocker; also inhibits store-operated Ca^2+^ entry, voltage-gated Ca^2+^ channels and potassium channels	15	NS^b^
Nicardipine	10	No effect	L-type VOCC blocker	18	NS^b^


*^a^Paired *t*-test, ^b^unpaired *t*-test.*

**Table 3:** UTP modulates BON cells MP by fine tuning of a number of ionic conductances.

Drug	Condition	Description	*p*-value	MP Depolarization (mV)	Increase in I_Ca_^2+^
UTP	100 μM (3 min)	P2Y_2,4,6_ endogenous agonist	n/a	5.94 ± 0.88 *n* = 17/34; (↑Ri)	*p* < 0.0001; *n* = 10 (V_m_ -20 to 40 mV)
UTPγS	30 μM (3 min)	P2Y_2,4_ agonist	n/a	n/a	*p* < 0.0001; *n* = 6 (V_m_ -30 to 20 mV)
UDP	100 μM (3 min)	P2Y_6_ agonist	*P* > 0.05	n/a	I_Ca_, *p* = NS; *n* = 8 Voltage dependence, *p* = 0.028; *n* = 8 (V_50_ -13.6 to -7.9)
Ca^2+^ free E.S. + EGTA	n/a	VOCa^2+^ channels inhibition	*P* > 0.05	4.66 ± 1.38, 3.70 ± 0.47 *n* = 9–10	n/a
Lanthanum^3+^	100 μ (10 min)	VOCa^2+^ channels blocker	*P* > 0.05	6.90 ± 1.05, 4.93 ± 1.07 *n* = 8–11	n/a
Thapsigargin	1 μM (10 min)	SERCA inhibitor	*P* > 0.05	4.90 ± 1.14, 6.18 ± 1.29 *n* = 11	n/a
U73122 vs. U73343	2.5 μM (5 min)	PLC antagonist	*P* < 0.05, χ^2^	# cells: 0/8, 5/12	n/a
GF109203X	0.5 μM (10 min)	PKC α,βI,βII, δ and ε inhibitor	*P* > 0.05	6.00 ± 1.00, 7.66 ± 1.45 *n* = 3	n/a
UTP vs. UTPγS	30 μM (3 min)	P2Y_2,4_ agonist	*P* > 0.05	7.16 ± 1.74, 6.83 ± 2.08 *n* = 6; (↑Ri)	n/a
UTPγS, +UTP	30 μM (3 min)	P2Y_2,4_ agonist	*P* < 0.05	8.30 ± 2.08, 0.40 ± 0.24 *n* = 5	n/a
UTP vs. XE-991	10 μM (10 min)	Kv 7.1,7.2 and 7.3 (KCNQ) blocker	*P* > 0.05	5.13 ± 0.64, 6.08 ± 0.82 *n* = 8–12; (↑Ri)	n/a
UTP, +XE-991	10 μM (10 min)	Kv7.1,7.2 and 7.3 (KCNQ) blocker	*P* < 0.05, χ^2^	# cells: 8/15, 2/17	n/a


**FIGURE 7:**
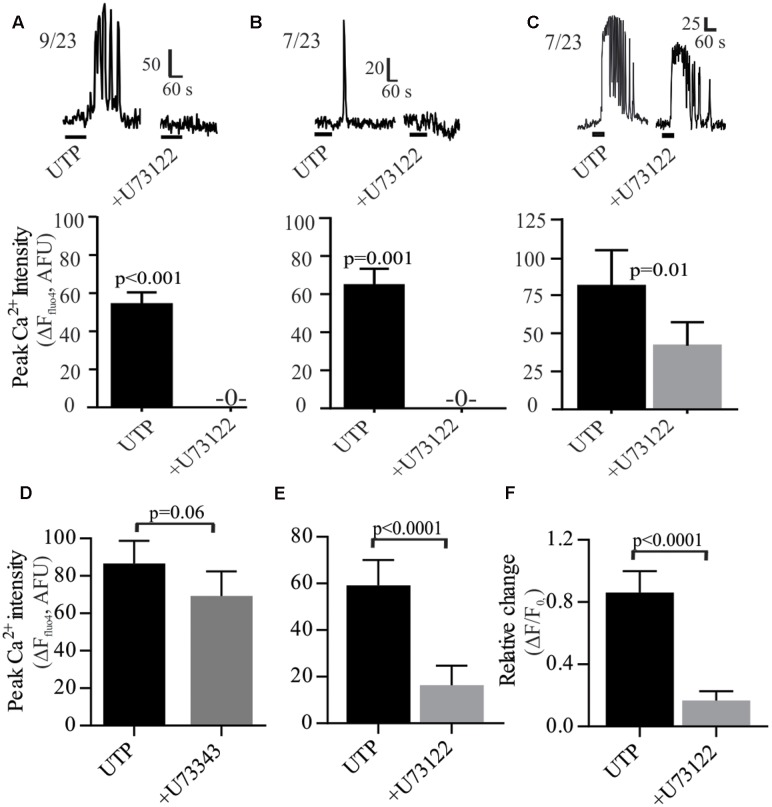
UTP-Ca^2+^ responses depend on the phospholipase C (PLC) signaling pathway in human BON. The PLC inhibitor U73122 (10 μM) abolished the Ca^2+^ responses induced by UTP **(A)** in cells with Ca^2+^ oscillations or **(B)** monophasic Ca^2+^ transients, **(C)** and reduced Ca^2+^ responses in other cells. Representative Ca^2+^ responses are shown in **(A–C)** and histograms associated with each type of response represent pooled data showing that U73122 can abolish or reduce Ca^2+^ responses. **(D–F)** Average population response showing that U73122 but not its inactive analog U73343, can block UTP-Ca^2+^ responses in BON (EC) cells. Cells were pre-incubated with U73122 or U73343 for 10 min.

As a GPCR, P2Y_4_ may couple to the G_q_ protein. The PLC inhibitor U73122 prevented the UTP – Ca^2+^ response in 69% of the cells and reduced the response in the remainder (**Figure 7**). Both types of responses, oscillations and monophasic Ca^2+^ transients to UTP were blocked by U73122 (**Figures 7A,B**). However, in a subset of cells, Ca^2+^ oscillations were only partially blocked by U73122 (**Figure 7C**). The effect of U73122 was selective for PLC since the inactive analog U73343 could not block UTP Ca^2+^ responses (**Figures 7D–F**).

**FIGURE 8:**
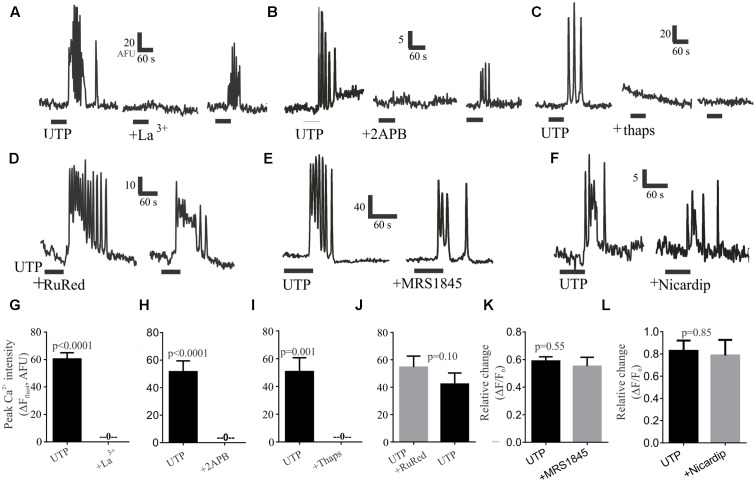
Post-receptor signaling pathways linked to UTP-Ca^2+^ responses in BON cells. Representative Ca^2+^ responses showing that **(A)** 100 μM La^3+^, **(B)** 10 μM 2APB or **(C)** 1 μM thapsigargin (thaps) can block UTP-induced Ca^2+^transients. In Contrast, **(D)** RuRed, **(E)** 20 μM MRS1845 or **(F)** 10 μM nicardipine are not effective in blocking UTP-induced Ca^2+^ transients. After washing-out the drug (15 min) a partial recovery of the UTP-response was seen for La^3+^ and 2APB but not for thapsigargin (thaps). **(G–L)** Pooled data for effects of each of the treatments illustrated in **(A–F)** indicating that La^3+^, 2APB or thaps can abolish Ca^2+^ transients (*p* < 0.001 for each) whereas RuRed, MRS1845 or Nicardipine did not significantly reduce UTP-induced Ca^2+^ transients (*p* > 0.05). **Table 2** provides detailed description of the inhibitors. The test group involved UTP application followed by UTP + inhibitor in the same cells (with a 10 min recovery time allowed between each drug application. A control group involved two consecutive applications of UTP (*p* > 0.05 between peaks in two consecutive applications, not shown).

To evaluate the role of external calcium on the rise of intracellular calcium, we tested the Ca^2+^ channel blocker lanthanum (La^3+^), which could abolish UTP – Ca^2+^ transients in 100% of cells in a partially reversible manner (**Figures 8A,G**). Next, to assess the role of intracellular calcium stores, we treated the cells with the IP3R antagonist 2APB, which could also abolish the UTP-Ca^2+^ transients, and the effect was partially reversible with washout of the drug (**Figures 8B,H**). In a similar way, the sarco/endoplasmic reticulum (SERCA) Ca^2+^ ATPase inhibitor thapsigargin, abolished all UTP-responses in an irreversible manner (**Figures 8C,I**).

To further dissect the signaling pathway, we used different treatments targeting several possibilities based on our previous results; however, they were without effect on UTP–Ca^2+^ responses (**Table 2**). These included treatment with a ryanodine receptor blocker (RuRed, **Figures 8D,J**), a selective inhibitor of store-operated Ca^2+^ entry (SOC channels, MRS1845; **Figures 8E,K**), a PI3K blocker (Ly294002, Supplementary Figure 3A), a PKC antagonist (GF109203X, Supplementary Figure 3B), and a SRC kinase antagonist (PP2, Supplementary Figure 3C), which did not affect the UTP – Ca^2+^ response. To further clarify the effect of La^3+^ on UTP-evoked oscillations, we tested inhibitors of other mechanisms that could potentially be involved in external calcium conductance. Therefore, the SOCE/TRPC channel blocker (SKF96365, Supplementary Figure 3D) and a blocker of the L-type voltage dependent Ca^2+^ channel, previously linked to 5-HT release (nicardipine, see **Figures 8F,L**) had no effect on UTP Ca^2+^ responses. Taken together, the data suggest that UTP Ca^2+^ responses are linked to a P2Y_4_/Gq/PLC/IP3/IP3R/SERCA-Ca^2+^pump – Ca^2+^ signaling pathway.

**FIGURE 9:**
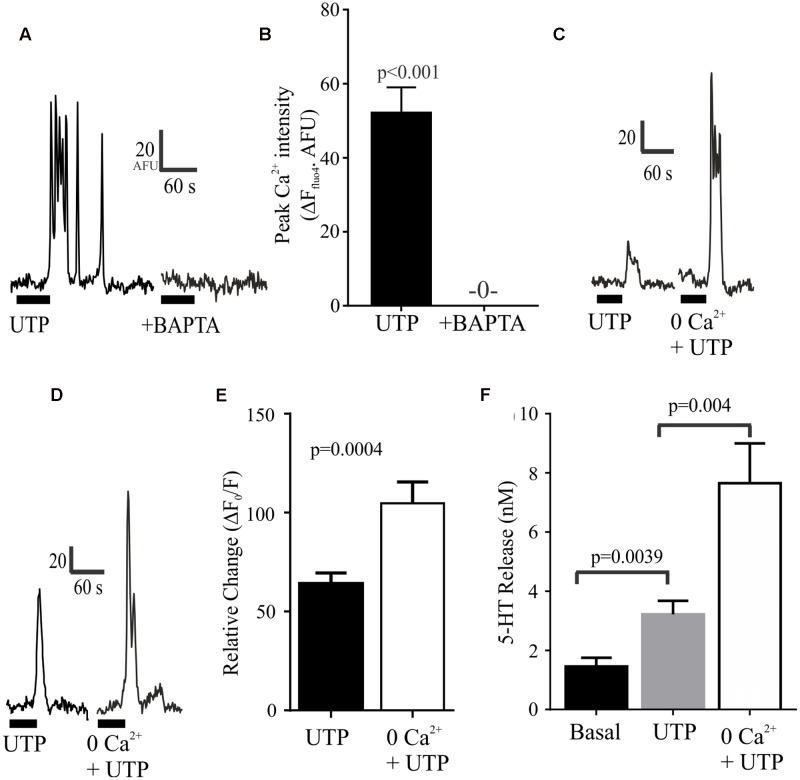
Depletion of intracellular or extracellular Ca^2+^ has different effects on UTP Ca^2+^ responses. **(A,B)** Chelating intracellular free Ca^2+^ levels with BAPTA-AM (50 μM) abolishes UTP-induced Ca^2+^ transients. **(C–E)** Ca^2+^ – free buffer (0.0 Ca^2+^ + EGTA) augments peak Ca^2+^ responses induced by UTP in a subset of cells. **(F)** In the absence of extracellular Ca^2+^ (Ca^2+^-free buffer), UTP is more effective in stimulating 5-HT release from BON cells (*N* = 12 cultures).

We further evaluated the effect of depleting intracellular or extracellular Ca^2+^ levels. We found that that these manipulations had opposite effects on UTP-evoked Ca^2+^ responses (**Figure 9**). On the one hand, the intracellular free Ca^2+^ chelator BAPTA-AM could abolish the UTP Ca^2+^ response in BON cells (**Figures 9A,B**). On the other hand, bathing the cells in Krebs buffer with 0.0Ca^2+^ + EGTA enhanced rather than suppressed UTP-evoked Ca^2+^ responses (**Figures 9C–E**) without affecting its duration (not shown). Finally, we evaluated whether or not this augmented intracellular calcium response may alter the magnitude of 5-HT release evoked by UTP and we found that, in BON cells, 0.0Ca^2+^ + EGTA in the extracellular bathing medium could enhance the UTP-evoked 5-HT release (**Figure 9F**).

### Molecular Signaling Pathways Linked to UTP-Induced MP Depolarization

**FIGURE 10:**
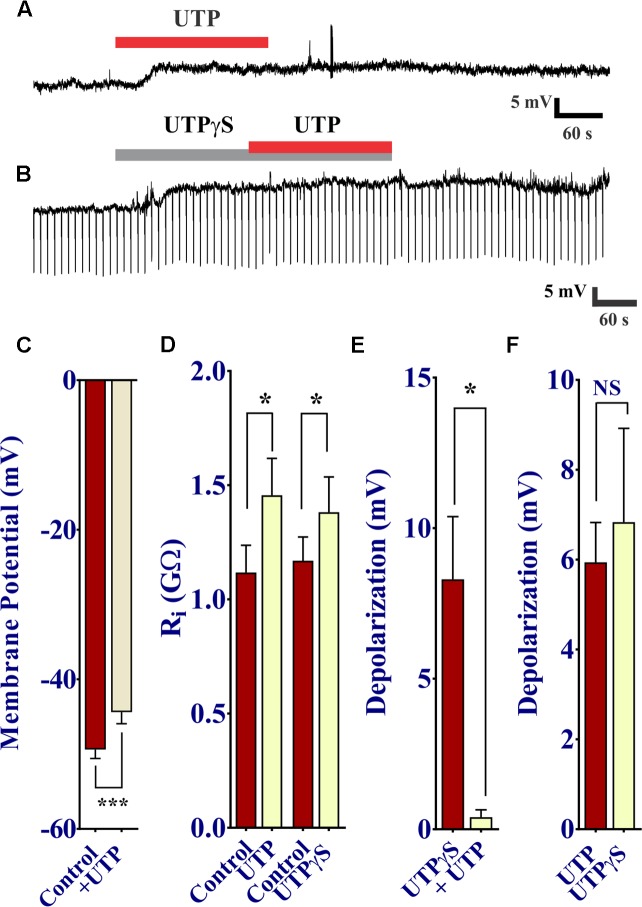
UTP depolarizes the membrane potential in human BON. **(A)** Acute application of 100 μM UTP causes V_m_ depolarization (upper trace, representative response), associated with an increase in cell input resistance (R_i_; **(B)**, lower panel; 1.12 ± 0.12 GΩ for control vs. 1.46 ± 0.16 GΩ for UTP, *p* = 0.01, *n* = 7; 1.12 ± 0.10 GΩ for control vs. 1.38 ± 0.15 GΩ for UTPγS, *p* = 0.038, *n* = 6). UTPγS mimicked UTP evoked V_m_ depolarization and if UTPγS is used to depolarize the cell, UTP no longer has any effect (lower trace). **(C)** UTP causes a significant depolarization of the membrane potential. **(D)** UTP or UTPγS causes a modest but significant increase in cell input resistance. Bars, SEM. Asterisk denotes statistical significance when analyzed with student’s *t*-test. **(E)** UTPγS induced V_m_ depolarization prevents the response to subsequent co-application of UTP (8.30 ± 2.08 mV for UTPγS and 0.40 ± 0.24 mV for UTP, *p* = 0.0165, *n* = 5). **(F)** UTP and UTPγS evoked a similar V_m_ depolarization (*p* > 0.05, NS; 5.94 ± 0.88 mV for UTP and 6.83 ± 2.09 mV for UTPγS, *n* = 17 and 6, respectively).

UTP was shown to cause a prominent increase in intracellular free Ca^2+^ levels, and therefore we sought to determine whether or not this increase in Ca^2+^ via P2Y receptor activation modulates the membrane potential of BON cells. To do so, we used perforated patch clamp to access and record membrane potential (V_m_) at rest and during drug stimulation. As expected, UTP induced a slow developing V_m_ depolarization in 50% of cells (17 of 34 BON cells). This effect peaked at ∼60 s of application and lasted several min after UTP washout (**Figures 10A,C** 5.94 ± 0.88 mV depolarization; *n* = 17). Surprisingly, UTP-evoked V_m_ depolarization was associated with a significant increase in R_i_ (**Figure 10D**, 1.11 ± 0.12 GΩ for control versus 1.46 ± 0.16 GΩ for UTP; *p* = 0.01, *n* = 7).

As our previous results demonstrated P2Y_4_ plays a major role in UTP responses in these cells, we tested UTPγS to determine the association of this receptor with the UTP-evoked V_m_ depolarization. In a similar manner, the P2Y_4_ agonist UTPγS caused a slow membrane depolarization equivalent to that of UTP (**Figure 10F**), associated with an increase in R_i_ (**Figure 10D**, 1.17 ± 0.10 GΩ for control versus 1.38 ± 0.15 GΩ for UTPγS; *p* = 0.038, *n* = 6); maximal depolarization was also reached at ∼60 s and lasted for several min after washing out UTPγS. Furthermore, activation of P2Y_4_ receptors by previous application of UTPγS prevented the UTP-evoked V_m_ depolarization (**Figures 10B,E**: 8.30 ± 2.08 mV for UTPγS and 0.40 ± 0.24 mV for UTP in the continued presence of UTPγ; *p* = 0.0165, *n* = 5). ATP caused a similar V_m_ depolarization to UTP, and prior application of ATP to depolarize the cell prevented any further response of UTP (Supplementary Figure 4). Therefore, activation of a P2Y_4_ receptor also causes V_m_ depolarization of BON cells.

To further dissect the intracellular pathway activated by UTP to cause V_m_ depolarization and in parallel with Ca^2+^ imaging studies, pharmacological studies with inhibitors were done. Data is summarized in **Table 3**.

**FIGURE 11:**
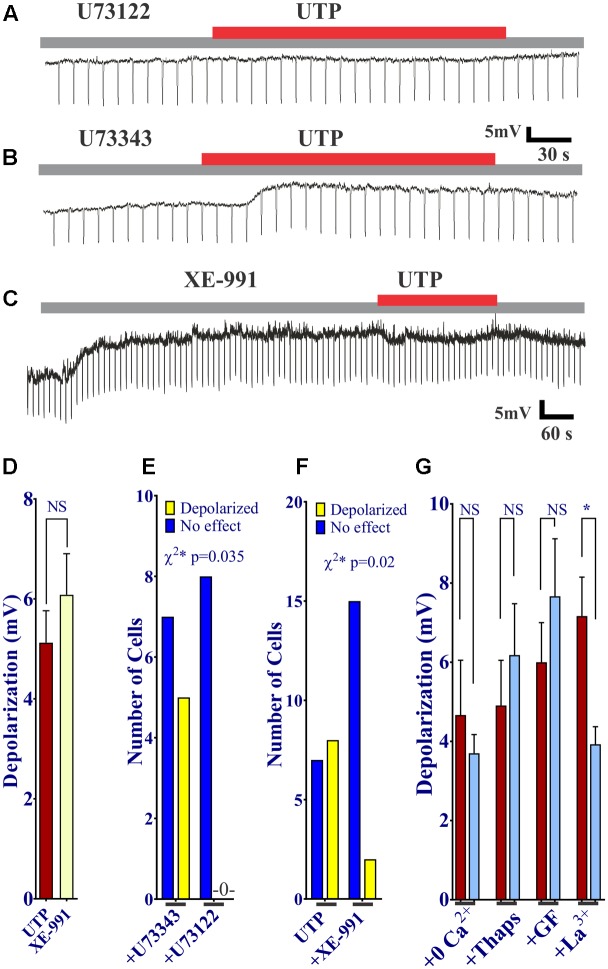
UTP evokes a K_v_7 and PLC-dependent V_m_ depolarization. **(A,B,D)** UTP depolarization was eliminated in the presence of a PLC inhibitor U73122 but not with the inactive compound U73343. **(C,E)** Blockade of K_v_ 7.1/7.2/7.3 potassium channels by XE-991 depolarizes the V_m_ and increases R_i_ in a similar manner to UTP (5.13 ± 0.64 mV for UTP vs. 6.08 ± 0.82 mV for XE-991). **(C,F)** Pre-incubation with 10 μM XE-991 reduced the number of cells responsive to UTP (*p* = 0.02). **(G)** Preincubation with La^3+^ inhibited the effect of UTP on membrane potential (^∗^*p* = 0.03). UTP-evoked V_m_ depolarization was not affected by treating cells with thapsigargin, GF109203X, and Ca^2+^ free external solution.

First of all, we sought to determine whether or not activation of P2Y was associated with PLC. The UTP induced V_m_ depolarization was completely abolished in the presence of the PLC inhibitor U73122 (**Figures 11A,E**), but not with the inactive compound U73343 (**Figures 11B,E**, 0 out 8 cells and 5 out of 12 cells, respectively, responded to UTP, *p* = 0.035). This may suggest a reduction in K^+^ conductance, an I_K_ current.

Since UTP-induced V_m_ depolarization was associated with an increase in R_i_ and previous studies reported UTP/P2Y_4_ can regulate potassium conductance via G_q_/PLC (type M currents) (Falkenburger et al., 2010), we wanted to assess if the effect of UTP in BON cells was associated with the modulation of the same conductance. Therefore, we tested XE-991, a selective Kv7.1/7.2/7.3 K^+^ channel blocker. Notably, blockage of type M K^+^ current with XE-991, which may be active at resting membrane potential (Wladyka and Kunze, 2006), mimicked UTP actions by causing V_m_ depolarization, and increased R_i_ (**Figures 11C,D,F**, 5.13 ± 0.64 mV depolarization for UTP versus 6.08 ± 0.82 mV depolarization for XE-991 alone). Furthermore, pre-incubation of BON cells with XE-991 significantly reduced the number of cells responsive to the application of UTP (8 out of 15 cells respond to UTP; 2 of 17 cells respond to UTP after XE-991 treatment, *p* = 0.02) (**Figures 11C,F**).

**FIGURE 12:**
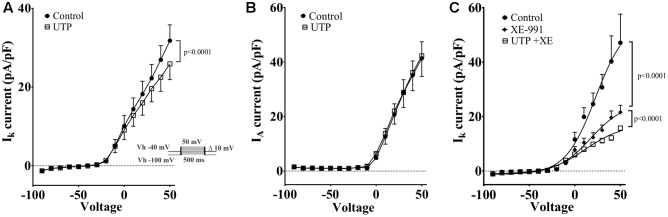
Cells treated with UTP exhibit a decrease in I_K_ potassium currents. Graphs show the mean I_K_ and I_A_ currents obtained by measuring the peak of each voltage step which were dissected by manipulating the holding potential. The I_A_ currents were determined by subtracting I_K_ currents from I_Tot_ currents. Insert shows the voltage steps protocol used to achieve voltage dependent potassium currents, V_h_ indicates holding potential. **(A)** Summary of data showing a decrease in I_K_ potassium currents initiated by UTP (*p* < 0.0001, *n* = 8). **(B)** I_A_ potassium currents were not significantly affected by UTP. **(C)** Shows a summary of results presenting a reduction in the evoked I_K_ currents (*p* < 0.0001, *n* = 3–5) in the presence of XE-991 alone and in combination with UTP. Symbols represent mean ± SEM.

K_v_7 potassium channel subunits can contribute to the generation of a voltage dependent outward current that does not inactivate in the presence of depolarizing pulses and they may participate actively in the control of the membrane potential (Schroeder et al., 2000). Therefore, we looked to further confirm the modulation of this conductance by UTP. We isolated voltage activated potassium currents, total K_v_ currents, and tested whether or not there was a modulation by UTP. As expected, acute UTP application (100 μM) reduced the K-type component (I_K_, **Figure 12A**, *p* < 0.0001, *n* = 8) without affecting the A-type outward potassium currents (I_A_, **Figure 12B**). A robust reduction of I_K_ currents was observed after incubation of the cells with the potassium channels blocker XE-991, generally used for selective blockage of subunits K_v_7.1/7.2/7.3 (**Figure 12C**, *p* < 0.0001, *n* = 3). However, UTP modulation of I_K_ currents persisted in the presence of XE-991 (**Figure 12C**, *p* < 0.0001, *n* = 5). These results suggest that besides the modulation of the potassium conductance involved in membrane potential control (i.e., carried by K_v_7.1/7.2/7.3 subunits), UTP may modulate an additional potassium conductance. The identity of this pathway/conductance was not considered further in our study. However, M-type currents seem to have small magnitude in BON cells, and we were unable to isolate them by voltage-clamp, and therefore, we were unable to further corroborate our findings on V_m_ with the Kv 7.1 antagonist XE-991. Therefore, it remains unresolved whether M-currents are being modulated by UTP. Furthermore, the effect of UTP on I_K_ does not account for its effects on V_m_ depolarization, since UTP reduced current at +50 mV and did not affect K^+^ currents at potentials close to the resting potential. We considered the possibility that more than one potassium conductance is being modulated by UTP.

### UTP Activates VOCC (I_Ca_ Currents)

**FIGURE 13:**
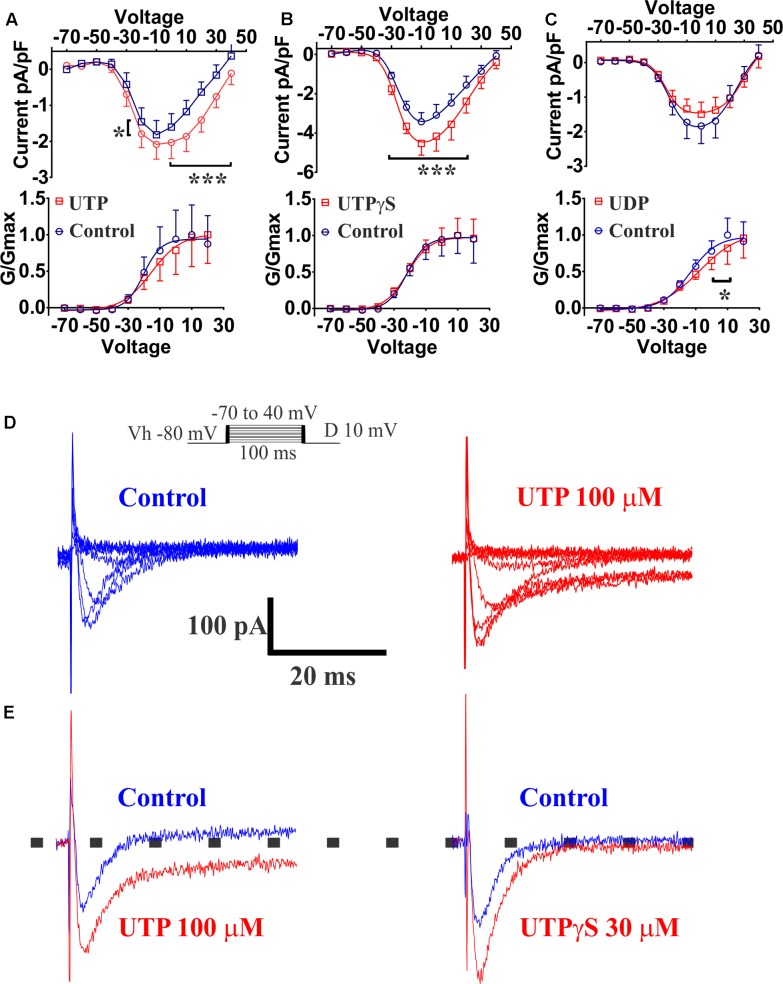
UTP increased currents carried by VOCC in a BON cell model of EC. Acute application of 100 μM UTP increased the magnitude of the currents carried by VOCCs while shifting the voltage dependence to the right. Acute application of UTPγS increased VOCC conductance (I_ca_) without affecting its voltage dependency. **(A–C)** Effect of bath application of UTP, UTPγS and UDP, respectively, are summarized as current/voltage curves (top panels). There was an increase in the I_ca_ currents after application of UTP (at V_m_ of –20, 0, to 40 mV, *p* < 0.0001, *n* = 10) and UTPγS (at Vm of –30 to 20, *p* < 0.0001, *n* = 6) but not in the presence of UDP (*n* = 8, *p* > 0.05). Bottom panels show voltage-response curves plotted as normalized conductance (*G*/*G*_max_) against voltage (mV), and adjusted by a Boltzmann dose-response equation. There was ∼5 mV shift in the voltage dependence when UTP was applied (–20.33 ± 5.611 mV control vs. –15.35 ± 8.045 mV UTP, *n* = 10, *p* = 0.1842) and a 5.7 mV shift when UDP was applied (–13.57 ± 4.22 mV control vs. –7.85 ± 7.99 mV UDP, *n* = 8, *p* = 0.0028). There was not any difference in voltage dependency with UTPγS (–21.94 ± 3.70 mV control vs. –21.81 ± 3.595 mV UTPγS, *n* = 6). **(D)** Representative current-trace for one cell before and after UTP stimulation (top panels up to 0 mV). **(E)** Representative control traces (blue) and after application (red) of UTP (left) and UTPγS (right). Persistence in the I_ca_ currents during the voltage steps was observed after stimulating the cells with UTP but not after UTPγS. Cells were depolarized from a holding potential of –80 mV with10 mV increasing voltage steps, which ranged from –70 to 40 mV with a duration of 100 ms (insert panel in **D**). Current amplitude was normalized to individual cell capacitance and showed as pA per pF. Data are plotted as mean ± SEM. Data were analyzed by two-way ANOVA, and statistical significances are denoted with asterisks (^∗∗∗^*p* < 0.001; ^∗^*p* < 0.05).

Previous reports had demonstrated that Ca^2+^ conductance via voltage-operated Ca^2+^ channels (VOCCs) could induce an increase in 5-HT release (Lomax et al., 1999). Our results indicated that the VOCC blocker La^3+^ abolished the UTP response observed with Ca^2+^ imaging, although an L-type Ca^2+^ channel blocker nicardipine did not inhibit the UTP response. Therefore, we employed the whole-cell patch clamp technique on BON cells to further assess VOCC activity in response to UTP. Ba^2+^ was used as a charge carrier in a standard solution free of Na^+^, and K^+^ channels were blocked by addition of TEA/4-AP and Cs in the electrode solution. After a 10 min stabilization period and bath solutions exchange, Ca^2+^ channels were voltage stimulated and evoked Ba^2+^ currents recorded before and after 3 min of UTP, UTPγS or UDP application. UTP significantly augmented the current density carried by VOCC (**Figure 13A**, at V_m_ of -20 mV, 0–40 mV, *p* < 0.0001; *n* = 10). The effect of UTP was reversible with a 3 min washout (not shown). UTP treated cells, also had an evident persistence of the currents during the application of voltage steps (**Figure 13E**). Interestingly, although not significant, UTP caused a shift in the VOCC voltage-dependence (**Figure 13A**, -20.33 ± 5.61 mV control vs. -15.35 ± 8.05 mV UTP, *n* = 10, *p* > 0.05).

The P2Y_2/4_ agonist UTPγS also augmented the currents carried by VOCC (**Figure 13B**, at V_m_ of -30–20 mV, *p* < 0.0001; *n* = 6); however, there was not any effect on the voltage dependency (**Figure 13**, control: -21.94 ± 3.70 mV versus UTPγS: -21.81 ± 3.595 mV, *n* = 6) and notably, cells treated with UTPγS did not show evidence of any change in the persistence of currents during stimulation with voltage steps as shown to occur with UTP treatment (**Figure 13E**). Interestingly, preferential P2Y_6_ stimulation by application of UDP, caused a statistically significant shift to the right (5.7 mV) in the VOCC voltage dependence (**Figure 13C**, control: -13.57 ± 4.22 mV versus UDP: -7.85 ± 7.99 mV; *n* = 8, *p* = 0.0028), and contrary to P2Y_4_ activation with UTPγS, there was not a significant change in the current density (**Figure 13B**, *n* = 8, *p* > 0.05). It is essential to note that no further analysis with a P2Y_2_ specific agonist was considered necessary as Ca^2+^ imaging studies ruled out any significant involvement of the receptor.

To determine whether or not the increase in VOCC conductance evoked by UTP was linked to the changes in MP, we performed a series of experiments in Ca^2+^ free external solution, and pre-incubating cells with La^3+^, a general blocker of VOCC. The latter intervention had an effect on the UTP-evoked V_m_ depolarization. V_m_ depolarization for UTP in normal Ca^2+^ solution was 4.67 ± 1.38 mV (*n* = 9) and 3.70 ± 0.47 mV (*n* = 10) for UTP in Ca^2+^ free medium (*p* > 0.05). For cells treated with La^3+^, V_m_ depolarization was 7.33 ± 1.05 mV for UTP (*n* = 12) and 3.93 ± 0.44 mV for UTP+La^3+^ (*n* = 7, *p* = 0.03) (**Figure 11G**). Furthermore, UTP-evoked V_m_ depolarization was not dependent of intracellular Ca^2+^ increase from intracellular stores, since, pre-incubation with a PKC inhibitor GF109203X (Toullec et al., 1991) and thapsigargin (Thaps) to deplete intracellular Ca^2+^ stores via activation of the sarco-endoplasmic reticulum Ca^2+^-ATPase (SERCA) were also not effective in reducing UTP-evoked V_m_ depolarization (**Figure 11G**, 4.91 ± 1.14 mV for UTP, *n* = 11; 6.18 ± 1.30 mV for UTP+Thaps, *n* = 11, *p* > 0.05).

These results suggest UTP activates simultaneously several parallel pathways that contribute to the activation of P2Y receptors. Whether or not UTP modulation of K^+^ conductance and VOCCs contribute to the modulation of 5-HT release is still to be confirmed.

## Discussion

ATP (or ADP) modulates mechanically-evoked 5-HT release via autocrine activation of stimulatory P2Y_1_, inhibitory P2Y_12_ purinergic pathways and ATP-gated P2X_3_-channels (Liñán-Rico et al., 2013). The current study expands our knowledge of purinergic signaling mechanisms by identifying uridine nucleotide receptors that are activated by UTP or UDP to induce a rise in intracellular free Ca^2+^ levels {[Ca^2+^]_i_}, Ca^2+^ oscillations, V_m_ depolarization and 5-HT release. These receptors are novel targets in EC cells for chemosensory regulation of gut reflexes.

### Role of P2Y_4_, P2Y_6_, P2Y_2_, Receptors in Ca^2+^ Signaling

UTP elicits a biphasic response in EC cells suggesting more than one receptor. At lower concentrations (EC_50_ = 11.4 μM), UTP activates both P2Y_4_ and P2Y_6_Rs (Abbracchio et al., 2006). The receptor activated at high concentrations is unknown. Functional P2Y_2_ receptors (P2Y_2_R, MRS2768) are rare in BON (EC) cells, and are not involved in Ca^2+^oscillations. UTP (P2Y_2,4,6_), UTPγS (P2Y_2,4_), UDP (P2Y_6_) or ATP induced Ca^2+^oscillations (or single Ca^2+^transients) in BON.

Overall, pharmacological and molecular analysis indicates that the predominant site is the P2Y_4_R in BON. However, expression of P2Y_4_R and P2Y_6_R is passage-dependent and therefore, definitive identification of the relative importance of these receptors awaits further study in freshly isolated human EC from surgical specimens that express both receptors.

Cells responded in the order of UTP/ATP > UTPγS (P2Y_2/4_) > UDP >> MRS2768 (P2Y_2_), BzATP (P2X_7_), α,β-MeATP (P2X_1,2/3,3_) > MRS2365 (P2Y_1_), MRS2690 (P2Y_14_), NF546 (P2Y_11_). P2X_1,2/3,3_R, P2X_7_R, P2Y_1_R, P2Y_11_R and P2Y_14_R play a minor role in chemosensory signaling. Agonist responses are consistent with the presence of multiple functional purinergic receptors. First, agonists with selectivity for different receptors had no effect, induced only a monophasic Ca^2+^response, or also had oscillatory responses. Second, different proportions of cells activated by UTP and ATP also responded to UTPγS (50% cells), UDP (30%), UTPγS/UDP (14%) or MRS2768 (<3%). Third, the profile of activity in response to consecutive applications of UTP, UTPγS, UDP (and ATP) is consistent with subsets of EC cells that express P2Y_4_, P2Y_6_ or both P2Y_4_ and P2Y_6_. In cells responding to UTP and ATP but no other agonists, different receptors are involved. ATP itself lucks intrinsic activity at human P2Y_4_R (Kennedy et al., 2000). P2Y_13_, P2X_4_, P2X_5_ or P2X_6_ are potential candidates for ATP (not UTP) responses (Communi et al., 1999; Zhang et al., 2002; Abbracchio et al., 2006; Kügelgen, 2006; Kügelgen and Bonn, 2008). UTP does not activate hP2Y_11_R (Morrow et al., 2014) and P2Y_11_ effects to NF546 (Meis et al., 2010) in BON were rare. The hP2Y_4_R forms stable dimers and transfected P2Y_4_R and P2Y_6_R proteins can associate with P2Y1,2,4,6,11 receptors (D’Ambrosi et al., 2006, 2007; Bilbao et al., 2012). Therefore, specific dimers could be responding to UTP and not UTPγS or UDP. This could also contribute to the biphasic nature of UTP responses at low or high micromolar levels.

Overall, the pharmacological data clearly show that subpopulations of cells exhibit responses to different sets of agonists. Subpopulations of responses suggest that UTP, ATP and UDP can activate different combinations of purinergic receptors in different cells, and therefore, subtypes of EC cells can be distinguished by their functional expression of purinergic receptors, including P2Y_4_, P2Y_6_, P2Y_2_, P2Y_4_/P2Y_6_, and other receptors not yet identified that respond to UTP and ATP.

### Post-receptor Ca^2+^ Signaling Pathways

Chelating intracellular free Ca^2+^levels prevented UTP – Ca^2+^responses indicating that the response is due to a rise in [Ca^2+^]_i_. Ca^2+^influx does not contribute to UTP-induced Ca^2+^responses (Nikodijevic et al., 1994), and suggests that the response may be a result of mobilization of Ca^2+^ from intracellular stores. Ca^2+^ free buffer augmented UTP-induced rise in [Ca^2+^]_i_ and 5-HT release, suggesting Ca^2+^ influx is an inhibitory modulatory mechanism in UTP responses. This was an unexpected finding and the mechanism involved remains unknown.

La^3+^ is an inhibitor of transmembrane Ca^2+^fluxes and a blocker of VOCCs (Block et al., 1998) and NSCC (Pena and Ordaz, 2008). In BON, La^3+^ was shown to block capsaicin-induced Ca^2+^ responses (Mergler et al., 2012). In our study, La^3+^could prevent the UTP-induced Ca^2+^ response suggesting that a NSCC or Ca^2+^ current through VOCCs is essential for the response. Nicardipine sensitive L-type VOCCs are not involved, although UTP activates other VOCCs (i.e., I_Ca_ currents).

The PLC inhibitor U73122 or an IP_3_R inhibitor 2APB could inhibit or abolish UTP responses in BON, indicating that the G_q_/PLC/IP_3_–Ca^2+^ signaling pathway is essential in triggering UTP Ca^2+^ responses. MRS1845 blockade of store-operated Ca^2+^entry (Harper et al., 2003) had no effect. 2APB could also modulate TRP channels (Alkhani et al., 2014), but a TRPC channel blocker SKF96365 that also inhibits store-operated Ca^2+^entry (SOCE) had no effect. PLC activation in non-excitable cells can cause release of Ca^2+^ from intracellular stores and activation of Ca^2+^ influx by means of Ca^2+^ release-activated channels (CRAC). The I_CRAC_ channel is not involved since SKF95365 had no effect on UTP responses (Kaznacheyeva et al., 2001). Other mechanisms were also excluded, including ryanodine-sensitive Ca^2+^pools and Kinases (PI3K, PKC, and SRC-K). The SERCA pump inhibitor thapsigargin (Tuluc et al., 2005) abolished UTP Ca^2+^responses, indicating that it is an essential mechanism.

### UTP – Induced V_m_ Depolarization, I_K_ and I_A_ Currents

UTP or UTPγS caused V_m_ depolarization in 50% of cells associated with increase in cell Ri, suggesting a closure of K^+^ channels. UTP did not affect the I_A_ current. Potassium channels, including K_v_ 7.1 have been implicated in regulation of secretion (Ullrich et al., 2005; Yamagata et al., 2011). UTP inhibits ATP sensitive and voltage-dependent K^+^ currents (I_K_, Welsh and Brayden, 2001). A K_v_ 7.1/7.2/7.3 K^+^ channel blocker XE-991 induced membrane depolarization of BON, mimicking the UTP-induced membrane depolarization and blocked UTP responses, implying that closure of IK_v_ 7.1/7.2/7.3 channels is linked to UTP depolarization. Desensitization to UTPγS prevents UTP responses, suggesting involvement of a P2Y_4_R. Findings with GF109203X or thapsigargin indicate that PKC or the SERCA mechanism is not involved in slow membrane depolarization. Overall, PLC inhibition blocked depolarization and Ca^2+^responses to UTP, whereas thapsigargin or zero Ca^2+^buffer had no effect on depolarization, indicating that V_m_ depolarization is not linked to Ca^2+^ responses. La^3+^ blocked UTP-evoked V_m_ depolarization, whereas zero Ca^2+^ buffer had no effect. This is explained by direct block of K^+^conductance (Type-M) by La^3+^ (Hirasawa et al., 2000).

When I_K_ currents were isolated, UTP reduced the overall prominent I_K_ currents. However, although a major component of the I_K_ currents could be blocked by a K_v_ 7.1,7.2,7.3 channel inhibitor XE-991 alone (Yeung and Greenwood, 2005), it did not block the effect of UTP on I_K_ currents, indicating that UTP can inhibit an I_K_ current that is insensitive to XE-991. Its identity remains unknown. XE-991 inhibits K_v_7 channels, but at the 10 μM concentration used, it may inhibit K_v_1.2/K_v_1.5 and K_v_2.1/K_v_9.3 channels (Zhong et al., 2010). However, at resting membrane potential of -50 to -60mV (-54 mV for BON), K_v_7 channels have an appreciable open probability that can be inhibited by XE-991 (Brown and Passmore, 2009; Mani et al., 2011). It has been shown K_v_7.2/7.3 channels are modulated by PLC/PIP_2_/IP_3_ in other cells. In fact, K_v_7 channels seem to require PIP2 to maintain open state (Zhang et al., 2003) and are negatively modulated by PIP2 membrane depletion (Hughes et al., 2007; Brown and Passmore, 2009; Falkenburger et al., 2010, 2013). Therefore, we suggest that UTP activation of PLC signaling leads to PIP2 depletion (a substrate for IP_3_ formation) to inactivate the K_v_7 channels, and closure of the channels leads to V_m_ depolarization. The effect of UTP on I_K_ currents seems independent of V_m_ depolarization. Specifically, the effect of UTP on K_v_ does not account for its effects on V_m_ depolarization, since UTP reduced current at +50 mV and did not affect K^+^currents at potentials close to the resting potential. We could not isolate other K_v_ currents (i.e., M-currents) in BON to test with UTP.

### Voltage-Dependent Ca^2+^ Currents (I_Ca_)

The fact that UTP and UTPγS but not UDP induced an increase of voltage-dependent Ca^2+^currents (I_Ca_) in BON suggests involvement of a P2Y_4_R in modulating VOCC. However, UTP-modulation of I_Ca_ was not linked to UTP-induced V_m_ depolarization, since zero Ca^2+^ buffer or nicardipine did not influence the response. The identity of the VOCC activated by UTP and its role in the regulation of 5-HT release remains unknown.

### Significance of Findings in BON and Translatability to EC Cells

As discussed in a recent review on sensory signaling in EC cells on 5-HT release, much of our knowledge and concepts of sensory signaling in EC cells (both mechanical and chemical signaling) comes from the human BON cell model of EC, although more recent work has included other cell lines, native EC cells and intact mucosa (Liñán-Rico et al., 2016). The pitfalls and use of BON or other cell lines, and human EC cells are described in detail in the review. Notably, some of the earlier publications on BON provided fundamental knowledge about mechanisms regulating EC cell function, i.e., mechanosensation (Kim et al., 2001a), glucose stimulation of 5-HT release (Kim et al., 2001b), purinergic autocrine regulation of 5-HT release in EC cells (Christofi et al., 2004a), and more recent studies on purinergic P2Y_1_, P2X_3_ and P2Y_12_ regulation of 5-HT release, also showing that P2X_3_ expression in native EC cells is severely down regulated in ulcerative colitis (Liñán-Rico et al., 2013). Data from BON on purinergic receptors are translatable to native EC cells (Chin et al., 2012; Liñán-Rico et al., 2013), and although our current study focused primarily on BON as a model of EC cells, we were able to confirm expression of the key receptor(s) in native EC cells and show that activation of uridine phosphate receptors by UTP stimulates 5-HT release. Furthermore, an unexpected finding worth further investigation is that calcium influx provides an inhibitory influence on 5-HT release. The data provide a basis for more complex and challenging experiments in native EC cells isolated from surgical specimens (Raghupathi et al., 2013), and selected studies on UTP regulation of 5-HT release in intact human surgical tissues with agonists and antagonists to confirm findings in a more physiologically intact environment.

**FIGURE 14:**
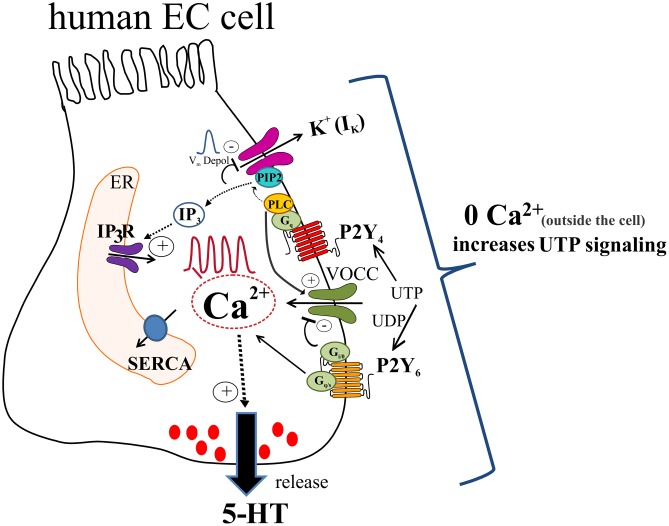
Working hypothesis of uridine nucleotide signaling in human EC cells lining the GI mucosa. UTP (or UDP) activate P2Y_4_ and P2Y_6_ receptors in EC cells to elevate intracellular free Ca^2+^ levels ([Ca^2+^]_i_) and generate Ca^2+^ oscillations or monophasic Ca^2+^ transients, and cause membrane depolarization. UTP acts via P2Y_4_ to stimulate a Gq/PLC-PIP2-IP3-IP3R/SERCA signaling pathway to elevate [Ca^2+^]_i_ associated with Ca^2+^ responses that triggers a Ca^2+^ dependent 5-HT secretion in EC cells. In addition, patch-clamp studies revealed that UTP increases I_Ca_ currents by activation of VOCCs via P2Y_4_; P2Y_6_ is presumed to have an inhibitory effect on VOCCs. As well, activation of P2Y_4_ causes a V_m_ depolarization that was independent of VOCC activation, but was eliminated by the K_v_7.1/7.2/7.3 channel inhibitor XE-991. We hypothesize that UTP depolarizes the cell by reducing a potassium conductance (not yet identified), which occurs as a consequence of depleting PIP2 from the membrane by its conversion to IP3 after PLC activation. V_m_ depolarization seems not linked to Ca^2+^ dependent 5-HT release but it may play a role in the fine tuning of the pathway. In a parallel way UTP reduced a component of I_K_ currents that was insensitive to XE-991, and the identity of the K_v_ channel remains unknown. This response does not explain the effect of UTP on V_m_ depolarization. A rise in intracellular free Ca^2+^ is required for 5-HT release. However, zero Ca^2+^ buffer augments both [Ca^2+^]_i_ inside the cell and 5-HT release. The mechanism for this paradoxical effect of Ca^2+^ influx is unknown. Concepts are based on data from a BON cell model of human EC cells. Isolated EC cells from human intestinal surgical specimens were used to confirm effect of UTP on 5-HT release and expression of P2Y_4_ and P2Y_6_ receptors.

Our working model of UTP signaling in EC cells is depicted in **Figure 14**. UTP elicits calcium oscillations via a G_q_/PLC/IP3/IP3R/SERCA Ca^2+^ pump signaling mechanism to stimulate 5-HT release through primarily a P2Y_4_R mechanism, although the P2Y_6_R may contribute. *Different combinations of receptors may be expressed in the same cells*, and subsets of EC cells can be distinguished by expressing functional P2Y_4_, P2Y_6_, P2Y_2_, P2Y_4_ and P2Y_6_ or other receptors. Activation of P2Y_4_R causes V_m_ depolarization dependent on I_K_ currents (not yet identified) and independent of VOCC/I_ca_, I_A_ or Ca^2+^oscillations. We hypothesize that UTP depolarizes the cell by reducing a potassium conductance through K^+^ channels, which occurs as a consequence of depleting PIP2 from the membrane by its conversion to IP_3_ after PLC activation. V_m_ depolarization seems not linked to Ca^2+^dependent 5-HT release but it may play a role in the fine tuning of the pathway. In a parallel way, UTP reduced a component of I_K_ currents that was insensitive to XE-991, and the identity of the specific K_v_ channel remains unknown. This effect of UTP on K_v_ channels does not explain its effect on V_m_ depolarization. UTP stimulates I_Ca_ currents by activating VOCCs not involved in V_m_ depolarization. A rise in intracellular free Ca^2+^is required for 5-HT release, although Ca^2+^ influx may attenuate 5-HT release. Ca^2+^influx through a La^3+^ – insensitive mechanism (since La^3+^ blocks Ca^2+^ responses) seems to provide ongoing inhibitory modulation of 5-HT release. It is not known whether all these mechanisms are linked to 5-HT release, and if they operate in normal EC cells. Monophasic and oscillatory Ca^2+^ responses may represent distinct receptors (or mechanisms).

Overall, UTP signaling is an important mechanism in regulation of 5-HT release from human EC cells, worth exploring in GI diseases or disorders associated with abnormal 5-HT signaling.

## Author Contributions

AL-R, study design, experiments, data analysis, interpretation and manuscript writing; contributed to Ca^2+^ imaging and molecular signaling studies, 5-HT release studies. FO-C, Study design, experiments, data analysis, interpretation and manuscript writing; contributed to patch-clamp and molecular signaling studies. AZ-A, Study design, experiments, data analysis, interpretation and manuscript writing; contributed to Ca^2+^imaging studies and concentration-response curve for UTP. MA, Contributed to molecular signaling studies and performed the western blot analyses; contributed to the results and discussion of the manuscript. JE, As an undergraduate student research assistant, he participated in all studies and di the immunohistochemical studies for P2Y_4_, 5-HT, TPH1 in BON or hEC cells. AH, Co-Investigator on NIH studies on purinergic regulation of EC cell release of 5-HT; GI Surgeon who was responsible for successful procurement of viable gut surgical specimens for isolating hEC cells for 5-HT release and IHC studies; contributed to study design, data analysis, and manuscript submission. ET, Contributed to qPCR analyses of P2Y_4_ and P2Y_6_ receptors, data analysis, interpretation and manuscript preparation. IG, Contributed to 5-HT release studies, IHC studies, data analysis, interpretation and manuscript preparation and all figures and illustrations. SB, co-mentor to AZ-A a postdoctoral fellow in the lab. Supported the overall study design, interpretation of data, and manuscript writing and submission. FC, Principal Investigator of the NIH funded study, and was responsible for the overall study design, conduct of the study, data analysis, interpretation, and manuscript writing and submission.

## Conflict of Interest Statement

The authors declare that the research was conducted in the absence of any commercial or financial relationships that could be construed as a potential conflict of interest.

## Abbreviations

[Ca^2+^]_i_: intracellular free calcium levels
CP: control immunogenic peptide
CRAC: calcium release activated channels
EC: enterochromaffin cell
GI: gastrointestinal
GPCR: G protein coupled receptor
hEC: human enterochromaffin cell
I_A_: type A voltage dependent potassium currents
I_ca2+_: Voltage dependent calcium currents
I_K_: type K voltage dependent potassium currents
K_v_: potassium currents
NSCC: non-selective cation currents
Ri: input resistance
RuRed: ruthenium red
SOCE: store operated Ca^2+^ entry
TPC: two-pore channel
VOCC: voltage operated calcium channel
