# Supplementary material for: UTP – Gated Signaling Pathways of 5-HT Release from BON Cells as a Model of Human Enterochromaffin Cells
Source: Front Pharmacol. 2017 Jul 13;8:429. doi: 10.3389/fphar.2017.00429 (PMC5508028; doi:10.3389/fphar.2017.00429)
Supplement: Supplementary file 1 [file Data_Sheet_1.pdf]

Supplementary Material

UTP – gated Signaling Pathways of 5-HT Release From Human Enterochromaffin Cells

Andromeda Liñán-Rico [1#], Fernando Ochoa-Cortes [1#], Alix Zuleta-Alarcon[1], Mazin Alhaj [1], Esmerina Tili[1, 2] Josh Enneking [1], Alan Harzman [3], Iveta Grants [1], Sergio Bergese[1], Fievos L. Christofi [1]**

Correspondence:

Fievos L. Christofi, Ph.D., AGAF

Fedias.Christofi@osumc.edu

Supplementary Figures

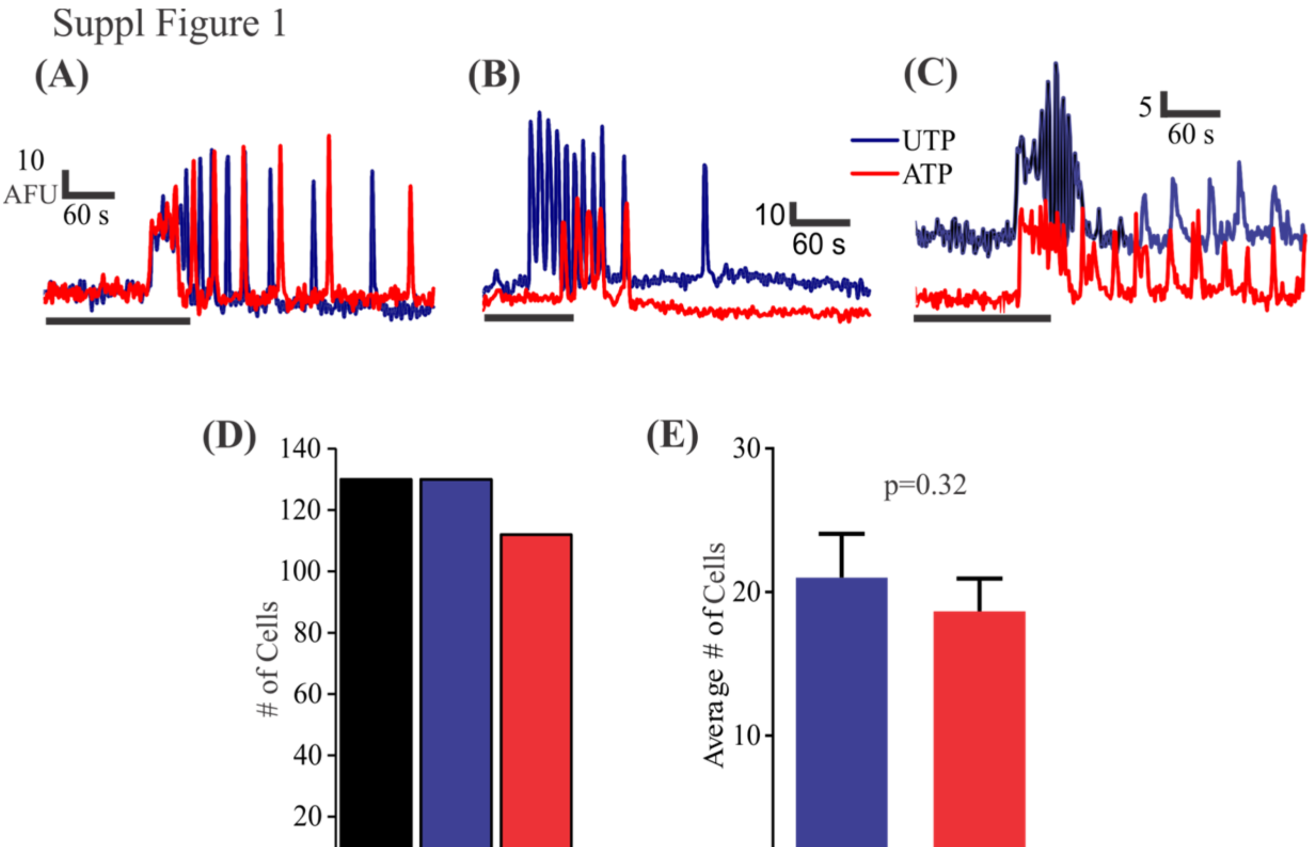

Suppl. Figure 1. Similar Ca^{2+} responses are induced by UTP and ATP in the same cells. (A-C) Representative traces of the Ca^{2+} transients for UTP and ATP responses in the same cell. (D) Total number of cells displaying UTP and ATP response; cells were first stimulated with UTP and then by ATP (100µM each) with a 10 minute washout. (E) Average number of cells responding to each agonist (n=6).

Suppl Figure 2

(A)

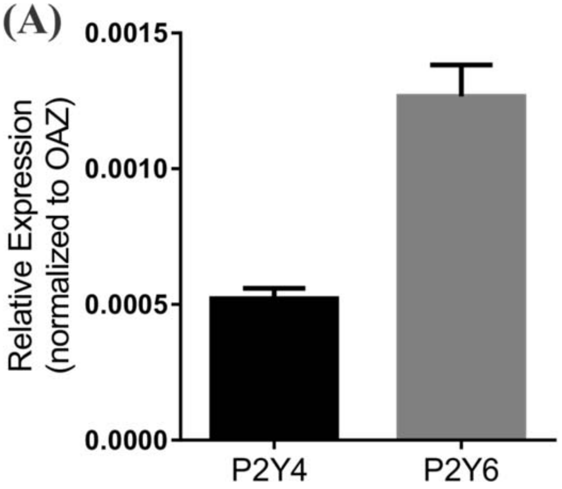

(B)

	UDP		UTPγS	
**Passage**	# of cells (vs UTP)	%	# of cells (vs UTP)	%
**<36**	53/82	64	60/113	53
**>43**	13/141	9	34/72	47

Suppl. Figure 2. mRNA expression levels of $P2Y_4$ and $P2Y_6$ is passage-dependent in BON cells. (A) qPCR showing the relative expression of $P2Y_4$ and $P2Y_6$ normalized to OAZ as 2^(ΔCt). The Ct values were 29 for $P2Y_4$ and 28 for $P2Y_6$ (n=5). BON cells for qPCR were tested at passage 36. (B) The table compares the proportions of responsive cells for UDP ($P2Y_6$ agonist) and UTPγS ($P2Y_4$ agonist) in relation to the passage number. The % of UTPγS-responsive cells does not change, whereas the % of UDP-responsive cells decreases dramatically in higher passages. Responses to agonist are dependent on relative expression of $P2Y_4$ and $P2Y_6$ receptors.

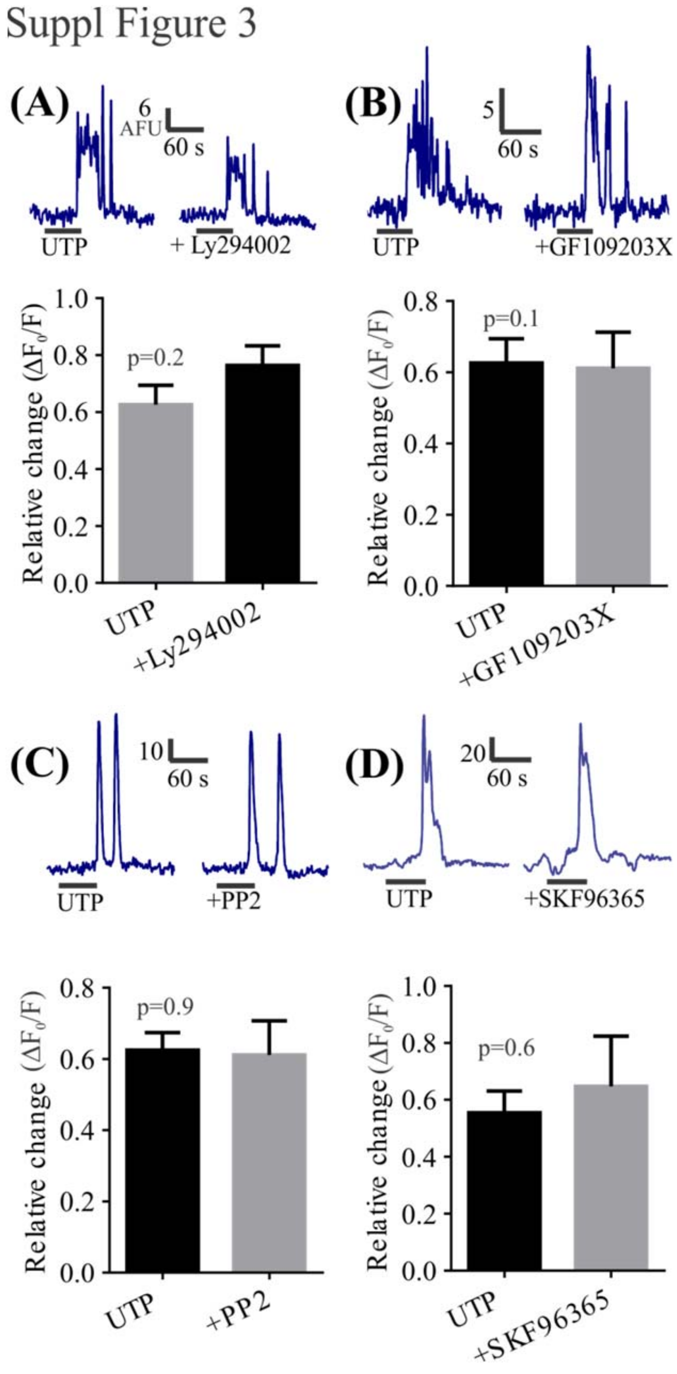

Suppl. Figure 3. Blocking PI3K, PKC, SRC or SOCE pathways did not affect the UTP Ca^{2+} response. Effect of (A) LY294002, (B) GF109203X, (C) PP2 and (D) SKF96365 in UTP Ca^{2+} responses expressed as relative change in Ca^{2+} intensity.

Suppl Figure 4

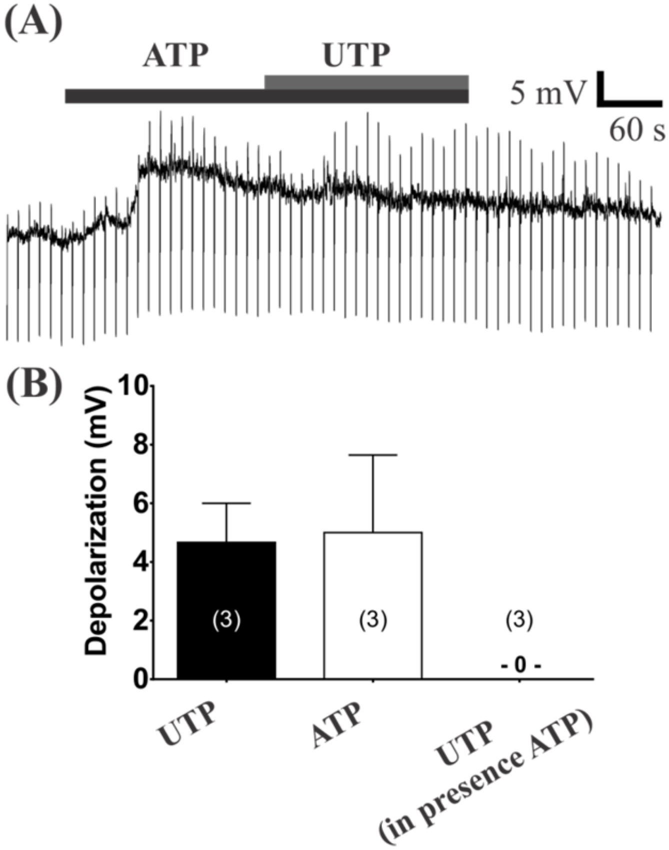

Suppl. Figure 4. ATP and UTP (100µM) caused MP depolarization associated with increase in membrane input resistance, denoted by higher hyperpolarization in response to electronic pulses. (A,B) ATP application evoked a response and prevented any additional UTP response. MP depolarizations; UTP $p>0.05$, UTP 4.67±1.33 mV, ATP 5.00±2.65 mV, and UTP (after ATP) 0.0±0.0mV; n=3.

1.2 Supplementary Table

Suppl. Table 1. Purinergic receptors involved in Ca^{2+} responses in BON cells

**Purine Agonist** (μM)	**ATP** (100)	**αβme-ATP** (10, 30)	**BzATP** (10,300)	**UTP** (100)	**UTPγS** (30)	**UDP** (100, 200)	**MRS2768** (10)	**NF546** (30μM)	**MRS2365** (1,5)	**MRS2690** (0.5)
**Target receptor**	P2X, P2Y	$P2X_{1,3}$ $P2X_{2/3}$	$P2X_7$, $P2Y_{11}$	$P2Y_{2,4,6}$	$P2Y_{2/4}$	$P2Y_6$	$P2Y_2$	$P2Y_{11}$	$P2Y_1$	$P2Y_{14}$
**Number of Ca^{2+} Responsive cells**	343	4/168 (ATP*) N=16	3/104 (ATP*) N=7	148/144 (ATP*) N=8	105/208 (UTP*) N=10	71/223 (UTP*) N=15	4/109 (UTP*) N=7	0/33 (ATP*) N=3	0/79 (ATP*) N=6	0/61 (ATP*) N=4

*Cells that were tested with a selective agonist had responses to ATP (or UTP) at the end of the experiment
